# Effect of Positron Emission Tomography Imaging in Women With Locally Advanced Cervical Cancer: A Randomized Clinical Trial

**DOI:** 10.1001/jamanetworkopen.2018.2081

**Published:** 2018-09-14

**Authors:** Lorraine M. Elit, Anthony W. Fyles, Chu-Shu Gu, Gregory R. Pond, David D’Souza, Rajiv Samant, Margaret Anthes, Gillian Thomas, Marc Filion, Julie Arsenault, Ian Dayes, Timothy J. Whelan, Karen Y. Gulenchyn, Ur Metser, Kavita Dhamanaskar, Mark N. Levine

**Affiliations:** 1 Juravinski Cancer Centre, Department of Obstetrics & Gynecology, McMaster University, Hamilton, Ontario, Canada; 2 Princess Margaret Cancer Centre, Department of Radiation Oncology, University of Toronto, Toronto, Ontario, Canada; 3 Ontario Clinical Oncology Group, Department of Oncology, McMaster University, Hamilton, Ontario, Canada; 4 London Regional Cancer Centre, Department of Oncology, University of Western Ontario, London, Ontario, Canada; 5 Ottawa Hospital Cancer Centre, Department of Medicine, University of Ottawa, Ottawa, Ontario, Canada; 6 Thunder Bay Regional Cancer Centre, Thunder Bay, Ontario, Canada; 7 Odette Sunnybrook Cancer Centre, Department of Radiation Oncology, University of Toronto, Toronto, Ontario, Canada; 8 Juravinski Cancer Centre, Department of Oncology, McMaster University, Hamilton, Ontario, Canada; 9 Hamilton Health Sciences, Department of Medicine, McMaster University, Hamilton, Ontario, Canada; 10 Hamilton Health Sciences, Department of Radiology, McMaster University, Hamilton, Ontario, Canada; 11 Princess Margaret Hospital, Department of Medical Imaging, University of Toronto, Toronto, Ontario, Canada

## Abstract

**Importance:**

In women with locally advanced cancer of the cervix (LACC), staging defines disease extent and guides therapy. Currently, undetected disease outside the radiation field can result in undertreatment or, if disease is disseminated, overtreatment.

**Objective:**

To determine whether adding fludeoxyglucose F 18 positron emission tomography–computed tomography (PET-CT) to conventional staging with CT of the abdomen and pelvis affects therapy received in women with LACC.

**Design, Setting, and Participants:**

A randomized clinical trial was conducted. Women with newly diagnosed histologically confirmed International Federation of Gynecology and Obstetrics stage IB to IVA carcinoma of the cervix who were candidates for chemotherapy and radiation therapy (CRT) were allocated 2:1 to PET-CT plus CT of the abdomen and pelvis or CT alone. Enrollment occurred between April 2010 and June 2014 at 6 regional cancer centers in Ontario, Canada. The PET-CT scanners were at 6 associated academic institutions. The median follow-up at the time of the analysis was 3 years. The analysis was conducted on March 30, 2017.

**Interventions:**

Patients received either PET-CT plus CT of the abdomen and pelvis or CT of the abdomen and pelvis.

**Main Outcomes and Measures:**

Treatment delivered, defined as standard pelvic CRT vs more extensive CRT, ie, extended field radiotherapy or therapy with palliative intent.

**Results:**

One hundred seventy-one patients were allocated to PET-CT (n = 113) or CT (n = 58). The trial stopped early before the planned target of 288 was reached because of low recruitment. Mean (SD) age was 48.1 (11.2) years in the PET-CT group vs 48.9 (12.7) years in the CT group. In the 112 patients who received PET-CT, 68 (60.7%) received standard pelvic CRT, 38 (33.9%) more extensive CRT, and 6 (5.4%) palliative treatment. The corresponding data for the 56 patients who received CT alone were 42 (75.0%), 11 (19.6%), and 3 (5.4%). Overall, 44 patients (39.3%) in the PET-CT group received more extensive CRT or palliative treatment compared with 14 patients (25.0%) in the CT group (odds ratio, 2.05; 95% CI, 0.96-4.37; *P* = .06). Twenty-four patients in the PET-CT group (21.4%) received extended field radiotherapy to para-aortic nodes and 14 (12.5%) to common iliac nodes compared with 8 (14.3%) and 3 (5.4%), respectively, in the CT group (odds ratio, 1.64; 95% CI, 0.68-3.92; *P* = .27).

**Conclusions and Relevance:**

There was a trend for more extensive CRT with PET-CT, but the difference was not significant because the trial was underpowered. This trial provides information on the utility of PET-CT for staging in LACC.

**Trial Registration:**

ClinicalTrials.gov Identifier: NCT00895349

## Key Points

**Question:**

In women with locally advanced carcinoma of the cervix who are candidates for chemotherapy and radiotherapy, does adding fludeoxyglucose F 18 positron emission tomography–computed tomography (PET-CT) to staging with CT of the abdomen and pelvis detect more extensive disease and influence therapy?

**Findings:**

In a randomized clinical trial, 44 of 112 patients receiving PET-CT compared with 14 of 56 patients receiving CT alone received more extensive chemotherapy and radiotherapy or palliative treatment, a nonsignificant difference. Five percent of patients in each group were treated with palliative intent.

**Meaning:**

In this trial among women with locally advanced carcinoma of the cervix, there was no significant difference between PET-CT plus CT vs CT alone, possibly because the trial was underpowered.

## Teaser

This randomized clinical trial compares the extent of treatment with chemotherapy and radiotherapy in women with locally advanced cervical cancer who have staging using fludeoxyglucose F 18 positron emission–computed tomography along with conventional staging with CT of the abdomen and pelvis vs those who have staging with CT alone.

## Introduction

Cervical cancer is the fourth most commonly diagnosed cancer in women, accounting for approximately 7.5% of female cancer deaths worldwide.^1^ When a patient presents with cancer of the cervix, the stage is determined to plan therapy. Approximately 40% of patients with cervical cancer present with locally advanced disease, defined as cancer that extends beyond the cervix to the vagina, parametrium, and/or lymph nodes.^2^ Treatment for locally advanced cancer of the cervix (LACC) is often with curative intent consisting of cisplatin chemotherapy and concurrent radiation therapy (CRT), external beam to the pelvis, and intracavitary brachytherapy. Staging is key to patient management as undetected disease outside the radiation field can result in undertreatment, or overtreatment if the undetected disease is disseminated. Historically, noninvasive testing using computed tomography (CT) with or without magnetic resonance imaging (MRI) was performed to determine whether cervical cancer was locally advanced.

Fludeoxyglucose F 18 positron emission tomography (PET) imaging is attractive because of the active uptake of radiolabeled fludeoxyglucose by tumor cells.^3,4,5,6,7^ Early studies of PET in patients with cervical cancer demonstrated that PET was superior to CT in detecting disease in pelvic and abdominal lymph nodes.^8^ These studies were small, were retrospective, and did not evaluate the utility of PET, ie, whether it led to a change in clinical management. The next step in the evolution of the test was that CT was combined with PET to provide anatomic structure to the functional imaging.^9^ However, there remained limited evidence of clinical benefit or improved outcome with PET-CT. Nonetheless, PET-CT began to be adopted in a number of jurisdictions.

Meanwhile, even though the preliminary studies of staging with PET-CT were encouraging, we felt that the evidence to support the routine adoption of PET-CT for staging in patients with LACC was insufficient to inform policy for the Ontario Ministry of Health. In 2009 we began planning a randomized trial evaluating the additional benefit of PET-CT to usual CT of the abdomen and pelvis in women with International Federation of Gynecology and Obstetrics (FIGO) stages IB to IVA carcinoma of the cervix who were candidates for CRT.

## Methods

### Patient Population

Eligible patients were women aged 18 years or older with newly diagnosed histologically confirmed (squamous, adenosquamous, or adenocarcinoma) FIGO stage IB-IVA carcinoma of the cervix^10^ being considered for curative treatment using concurrent chemotherapy and pelvic radiotherapy. Women not suitable for primary surgery because of comorbidities were also considered eligible. Full details are available in the trial protocol in Supplement 1.

Patients were excluded for Eastern Cooperative Oncology Group performance status greater than 2; other cervical cancer histologic types (eg, neuroendocrine); carcinoma of the cervical stump; having undergone a whole-body PET-CT scan within the last 6 months; contraindications for fludeoxyglucose F 18 PET-CT or CT of the abdomen or pelvis; inability to lie supine for imaging; contraindications to radiotherapy or cisplatin chemotherapy; inadequate bone marrow, renal, or hepatic function; history of another invasive malignant tumor within the previous 5 years with the exception of nonmelanoma skin cancer; known pregnancy or lactating; or inability to complete the study or required follow-up.

Patients were recruited at 6 regional cancer centers in Ontario, Canada, where CRT is delivered. The PET-CT scanners were located at 6 academic institutions (Ottawa Hospital, Ottawa; Princess Margaret Cancer Centre, Toronto; St. Joseph’s Hospital, Hamilton; St. Joseph’s Health Care, London; Sunnybrook Cancer Centre, Toronto; and Thunder Bay Regional Hospital, Thunder Bay). Institutional review boards at each center and Health Canada approved the study protocol. All patients provided written informed consent. The study followed the Consolidated Standards of Reporting Trials Extension (CONSORT Extension) reporting guideline.

### Randomization and Study Interventions

Initial assessment was performed, including physical examination and cancer staging according to FIGO 2009 criteria. Chest radiography or chest CT scan and blood tests were required within 28 days prior to randomization. Patients completed quality-of-life (QOL) questionnaires, and radiation oncologists were asked about the initial radiation treatment plan. Patients were then randomized centrally through the Ontario Clinical Oncology Group located in Hamilton using a web-based interactive registration and randomization system. A computer-generated permuted-block randomization schedule was used, with stratification for FIGO stage (IB-IIA vs IIB-IVA), prerandomization pelvic MRI, prerandomization CT scan, and treatment center. Allocation occurred in a 2:1 fashion to imaging with CT of the abdomen and pelvis plus PET-CT vs standard imaging.

### Abdominal and Pelvic CT Scans

Patients in both groups had a conventional CT of the abdomen and pelvis. These were performed using local imaging protocols and were read by the center radiologists according to the standard of care.

### Examination With PET-CT Imaging

The PET-CT examination was performed according to standard institutional protocols after a fast of 6 hours. Blood glucose level was required to be less than 180.18 mg/dL (to convert to millimoles per liter, multiply by 0.0555) prior to intravenous administration of fludeoxyglucose F 18 (5 MBq/kg [0.14 mCi/kg], not exceeding 550 MBq [14.9 mCi]). A low-dose CT used for attenuation correction preceded PET acquisition, and a whole-body PET-CT scan in supine position was obtained from the skull base to the upper thighs. Subsequently, a contrast-enhanced CT of the abdomen and pelvis was acquired. Imaging standards for CT were identical to those obtained for patients in the CT group. In some cases, this was performed on a separate CT scanner. Patients were required to undergo PET-CT within 6 weeks of randomization.

The PET-CT image was interpreted by the nuclear medicine physician at the study site and a second nuclear medicine physician from an external center. Any major discrepancies were resolved by consensus. Each focus of fludeoxyglucose uptake on the PET-CT scan was interpreted using a 5-point ordinal scale with 0 indicating normal; 1, probably normal; 2, equivocal; 3, probably abnormal; and 4, definitely abnormal).^9^ For the purpose of analysis, the probably abnormal and definitely abnormal categories were considered to be positive for the presence of cancer. Interpretation of lymph node metastases was based on visual criteria. Specifically, lymph nodes 1 cm or less in diameter were considered positive if there was fludeoxyglucose uptake greater than that of the surrounding background, and lymph nodes larger than 1 cm in diameter were considered positive if uptake was greater than blood pool activity. Measurement of maximum standard uptake value (SUV) normalized to body mass was obtained from all primary cervical tumors. When the Ontario Clinical Oncology Group program for the evaluation of PET in oncology commenced in 2002, a quality assurance program was put in place to establish standards for PET scanners, radioisotopes, and interpretation of scans.^11^

### Chemotherapy and Radiation

Guidelines for the extent of radiotherapy based on the imaging results are provided in eTable 1 in Supplement 2. Treatment plans were ultimately determined by the treating investigator following discussion with the patient. Chemotherapy was provided as cisplatin, 40 mg/m^2^ weekly for a total of 5 to 6 cycles during radiation. Whole-pelvic external radiation included 45 to 50 Gy (to convert to rad, multiply by 100) in 1.8 Gy per fraction for 25 fractions over a period of 5 weeks. Intracavitary brachytherapy of 35 to 40 Gy was given in 1 to 2 implants of low dose rate or pulse dose rate, or 24 to 30 Gy in 3 to 5 fractions of high dose rate brachytherapy. Parametrial boost 5 to 10 Gy, 1.8 to 2 Gy, and 3 to 5 fractions over 3 to 5 days to involved parametria was added if the total dose to the sidewall was less than 60 Gy. Overall treatment duration was not to exceed 8 weeks.

Following completion of the study, an independent blinded expert panel of 2 oncologists (I.D. and J.A.) centrally adjudicated all treatment plans. Discrepancies were resolved by consensus.

### Follow-up

On completion of treatment, the patient was seen every 3 months for 2 years, then every 4 months for 1 year, and then every 6 months for 2 years. Disease status, Eastern Cooperative Oncology Group performance status, and QOL information were captured every 6 months for 2 years and then annually for 3 years.

### Outcomes

The primary outcome was the treatment delivered to the patient: standard pelvic CRT with curative intent; more extensive CRT with curative intent (ie, extended field radiotherapy [EFRT]), or therapy with palliative or noncurative intent. Standard pelvic CRT with curative intent was defined as a 4-field plan to a superior border of L5 to S1.^12,13^ Any patient who received radiotherapy to para-aortic nodes (L1-L3) or common iliac nodes (L4-L5) was deemed as having more extensive CRT with EFRT. Prespecified secondary outcomes included event-free survival (EFS), overall survival (OS), prognostic value of the SUV of the cervix, and QOL. Quality of life was assessed with the general and cervix cancer–specific symptom tools (European Organisation for Research and Treatment of Cancer QLQ-c30 [version 3] and QLQ-CX24).^14^ Health utility was assessed using the EQ-5D health questionnaire.^15^

### Statistical Analysis

The primary outcome for analysis was treatment delivered (defined as a binary outcome of standard CRT with curative intent vs EFRT or treatment with palliative intent). The primary analysis was based on a logistic regression analysis, with adjustment for stratification factors. Secondary analyses explored the effect of imaging on EFS and OS. Event-free survival was calculated from the date of randomization to the date of objective disease recurrence, progression (positive result on a biopsy or radiologic imaging), or death due to any cause. Overall survival was calculated from the date of randomization to the date of death due to any cause. Patients without an EFS or OS event were censored on the last date they were confirmed to be event free. Time-to-event outcomes were examined using Kaplan-Meier methods and compared using Cox proportional hazards regression models adjusted for stratum. Because of nonnormality of SUV, a logarithmic transformation was performed to normalize the variable. The prognostic ability of the log-SUV was evaluated for OS and EFS. We calculated 95% confidence intervals for differences in proportions using the Wald approximation. A modified intention-to-treat principle was used and decided on prior to any analysis, whereby any patient who was randomized but did not undergo imaging was omitted from analysis. The trial protocol in Supplement 1 provides full details.

All analyses were 2-sided and a *P* value of .05 or less was considered statistically significant. All analyses were performed using SAS statistical software version 9.2 (SAS Institute Inc), and plots constructed using R statistical software v3.1.2 (R Project for Statistical Computing).

### Sample Size

A trial with a sample size of 288 (192 PET-CT and 96 CT abdomen-pelvis) was targeted, resulting in greater than 90% power to detect a 20% difference in treatment with EFRT or palliation (eAppendix in Supplement 2).

## Results

### Patient Population

**Figure 1.**
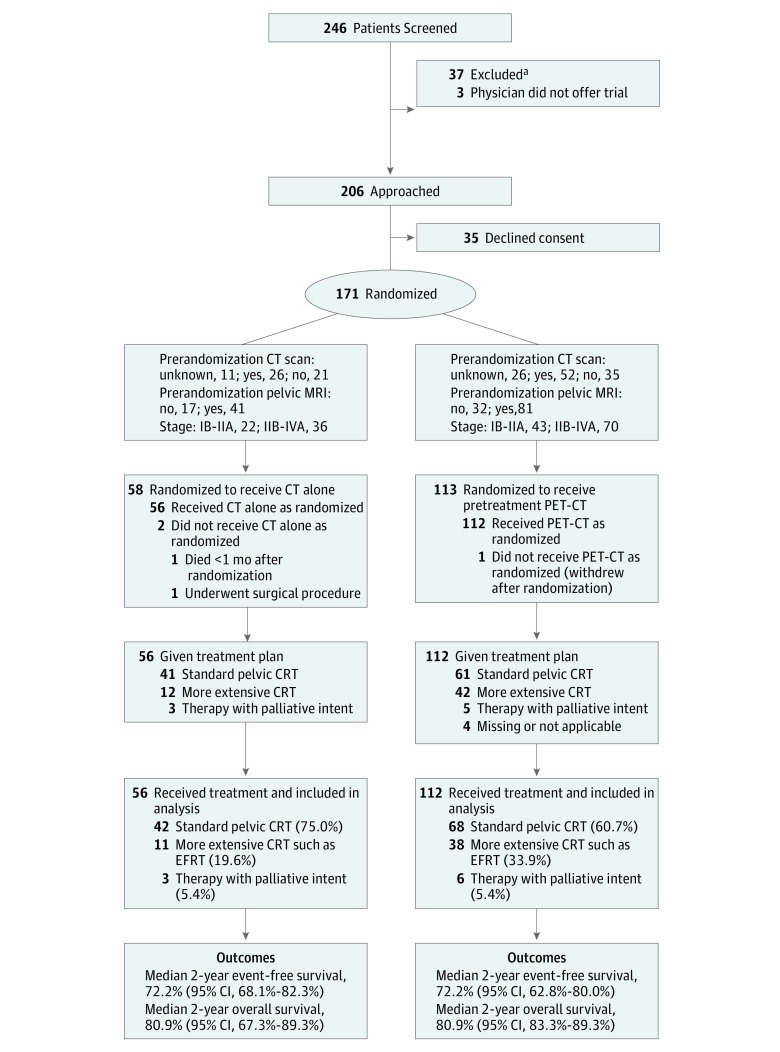
CONSORT Diagram ^a^Exclusion criteria (can be multiple per patient) were Eastern Cooperative Oncology Group performance status less than 2 (n = 2), other cervical cancer type (n = 9), carcinoma of the cervical stump (n = 2), prior hysterectomy (n = 4), already undergone positron emission tomography–computed tomography (PET-CT) (n = 1), previous CT of abdomen or pelvis (n = 8), inability to lie supine (n = 4), contraindication to radiotherapy (n = 1), contraindication to cisplatin (n = 2), inadequate bone marrow function (n = 4), inadequate renal function (n = 1), inadequate hepatic function (n = 2), history of other malignancy (n = 1), other medical condition (n = 3), known pregnancy or lactating (n = 4), and inability to complete study (n = 2). CRT indicates chemotherapy and radiation therapy; EFRT, extended field radiotherapy; and MRI, magnetic resonance imaging.

Between April 2010 and June 2014, 246 patients were screened and 208 eligible patients were approached for participation. Because of slower-than-expected accrual, the trial was stopped in June 2014 after a total of 171 patients were entered, of whom 113 were allocated to the PET-CT group and 58 to the CT group (Figure 1). In the PET-CT group, 1 patient withdrew the day after randomization. In the CT group, 1 patient died within a month of randomization and was later found to have stage IV disease. One patient decided to have surgery instead of CRT based on findings from her prerandomization MRI. None of these patients had study imaging and all are excluded from further analyses. The 2 study groups were reasonably comparable for baseline characteristics (Table 1). Mean (SD) age was 48.1 (11.2) years in the PET-CT group and 48.9 (12.7) years in the CT group.

**Table 1.** Baseline Characteristics

Characteristic	No. (%)	
	CT of Abdomen and Pelvis (n = 58)	CT of Abdomen and Pelvis Plus Whole-Body PET-CT (n = 113)
Stratum		
Pelvic MRI		
No	17 (29.3)	32 (28.3)
Yes	41 (70.7)	81 (71.7)
Stage		
IB-IIA	22 (37.9)	43 (38.1)
IIB-IVA	36 (62.1)	70 (62.0)
Prerandomization CT		
Prior to amendment	11 (19.0)	26 (23.0)
No	21 (36.2)	35 (31.0)
Yes	26 (44.8)	52 (46.0)
Demographic characteristics		
Study center		
Juravinski	19 (32.8)	32 (28.3)
London	7 (12.1)	14 (12.4)
Sunnybrook-Odette	5 (8.6)	5 (4.4)
Ottawa	7 (12.1)	17 (15.0)
Thunder Bay	5 (8.6)	9 (8.0)
University Health Network	15 (25.9)	36 (31.9)
Age, y		
Mean (SD)	48.1 (11.2)	48.9 (12.7)
Median (range)	47.1 (27.9-76.0)	47.8 (24.4-83.8)
Eastern Cooperative Oncology Group performance status		
0	43 (74.1)	77 (68.1)
1	14 (24.1)	34 (30.1)
2	1 (1.7)	2 (1.8)
Height, mean (SD), cm	163.2 (8.1)	162.0 (7.1)
Weight, mean (SD), in kg	75.8 (22.4)	70.3 (15.4)
Body mass index^a^		
Mean (SD)	28.2 (7.2)	26.8 (5.4)
Median (range)	28.1 (16.5-44.2)	25.8 (18.1-46.5)
Relevant comorbidities		
No	31 (53.5)	63 (55.8)
Yes	27 (46.6)	50 (44.3)
Tumor characteristics		
International Federation of Gynecology and Obstetrics stage		
IB1	3 (5.2)	11 (9.7)
IB2	14 (24.1)	27 (23.9)
IIA1	1 (1.7)	3 (2.7)
IIA2	2 (3.5)	1 (0.9)
IIB	28 (48.3)	45 (39.8)
IIIA	1 (1.7)	2 (1.8)
IIIB	8 (13.8)	24 (21.2)
IVA	1 (1.7)	0
IB-IIB	48 (82.8)	87 (77.0)
IIIA-IVA	10 (17.2)	26 (23.0)
Time from diagnosis to registration, median (range), mo	0.4 (−0.2 to 3.1)	0.2 (−0.1 to 4.5)
Radiology imaging		
Chest radiography		
No	21 (36.2)	37 (33.3)
Yes	37 (63.8)	74 (66.7)
CT of thorax		
No	44 (75.9)	73 (64.6)
Yes	14 (24.1)	39 (34.5)
Missing	0	1 (0.9)
MRI of pelvis		
No	17 (29.3)	36 (31.9)
Yes	41 (70.7)	77 (68.1)
CT of abdomen		
No	27 (46.6)	51 (45.1)
Yes	31 (53.5)	62 (54.9)

Abbreviations: CT, computed tomography; MRI, magnetic resonance imaging; PET, positron emission tomography.

^a^ Calculated as weight in kilograms divided by height in meters squared.

### Primary Outcome

Of 112 patients who underwent PET-CT imaging, 68 (60.7%) were treated with standard pelvic CRT of curative intent, 38 (33.9%) received more extensive CRT, and 6 (5.4%) received therapy with palliative intent (Table 2). In contrast, of 56 patients who underwent imaging with standard CT, 42 (75.0%) received standard pelvic CRT, 11 (19.6%) received more extensive CRT, and 3 (5.4%) received therapy with palliative intent (Table 2). Therefore, there were 44 patients in the PET-CT group (39.3%) who received more extensive CRT of curative intent or palliative treatment compared with 14 patients in the CT group (25.0%). Adjusting for baseline stratum, this difference was not statistically significant (odds ratio [OR], 2.05; 95% CI, 0.96-4.37; *P* = .06) using logistic regression.

**Table 2.** Primary Outcome

Treatment	No. (%)	
	CT of Abdomen and Pelvis (n = 56)	CT of Abdomen and Pelvis Plus Whole-Body PET-CT (n = 112)
Standard pelvic chemotherapy and radiotherapy	42 (75.0)	68 (60.7)
More extensive chemotherapy and radiotherapy	11 (19.6)	38 (33.9)
Palliative intent	3 (5.4)	6 (5.4)
More extensive chemotherapy and radiotherapy or palliative intent	14 (25.0)^a^	44 (33.9)^a^

Abbreviations: CT, computed tomography; PET, positron emission tomography.

^a^ Odds ratio, 2.05; 95% CI, 0.96-4.37; *P* = .06.

Twenty-four patients in the PET-CT group (21.4%) received EFRT to para-aortic nodes and 14 (12.5%) to common iliac nodes compared with 8 (14.3%) and 3 (5.4%), respectively, in the CT group (OR, 1.64; 95% CI, 0.68-3.92; *P* = .27). The rate of potentially curative EFRT to para-aortic nodes was increased by 7.1% in the PET-CT group. Three additional patients allocated to PET-CT and 2 additional patients allocated to CT alone had disseminated disease (stage IV) detected by the imaging test.

### Imaging Tests and Lymph Nodes

Fifty-eight of 103 patients in the PET-CT group (56.3%) had normal scan results. In the remaining patients, 18 (17.1%) had only abnormal pelvic nodes and 28 (27.7%) had abnormal common iliac or para-aortic nodes (eTable 2 in Supplement 2). Of the 55 patients in the control group for whom diagnostic CT results were available, 34 patients (61.8%) had normal scan results, 13 patients (23.6%) had only abnormal pelvic nodes, and 8 patients (14.5%) had abnormal common iliac or para-aortic nodes (eTable 3 in Supplement 2). Comparison of the rates of abnormal common iliac or para-aortic nodes between groups was not statistically significantly different.

### Imaging Tests and Treatment Delivered

In the 58 patients with normal PET-CT scan results, 12 received more extensive radiation and 1 received palliative treatment (eTable 3 in Supplement 2). For the 17 PET-CT patients with only pelvic nodes abnormal, 11 received standard pelvic radiation, 5 received more extensive radiation, and 1 underwent palliative treatment. For the 28 patients with abnormal common iliac or para-aortic nodes, 7 received standard pelvic radiation.

In the CT group, 3 of 34 patients received more extensive or palliative therapy even though the CT scan results were normal, and 2 of 8 patients received standard therapy even though the common iliac or para-aortic results were abnormal (eTable 3 in Supplement 2).

### Relationship Between Diagnostic CT and PET-CT

An exploratory analysis was conducted to examine a potential association between the presence of pelvic nodes on the diagnostic CT result and the result of the subsequent PET-CT. Twelve of 36 patients (33.3%) with abnormal pelvic nodes on CT had positive para-aortic nodes on PET-CT. In contrast, 8 of 65 patients (12.3%) with normal pelvic nodes on CT had a positive para-aortic node (OR, 3.56; 95% CI, 1.29-9.82; *P* = .01).

### Secondary Outcomes

**Figure 2.**
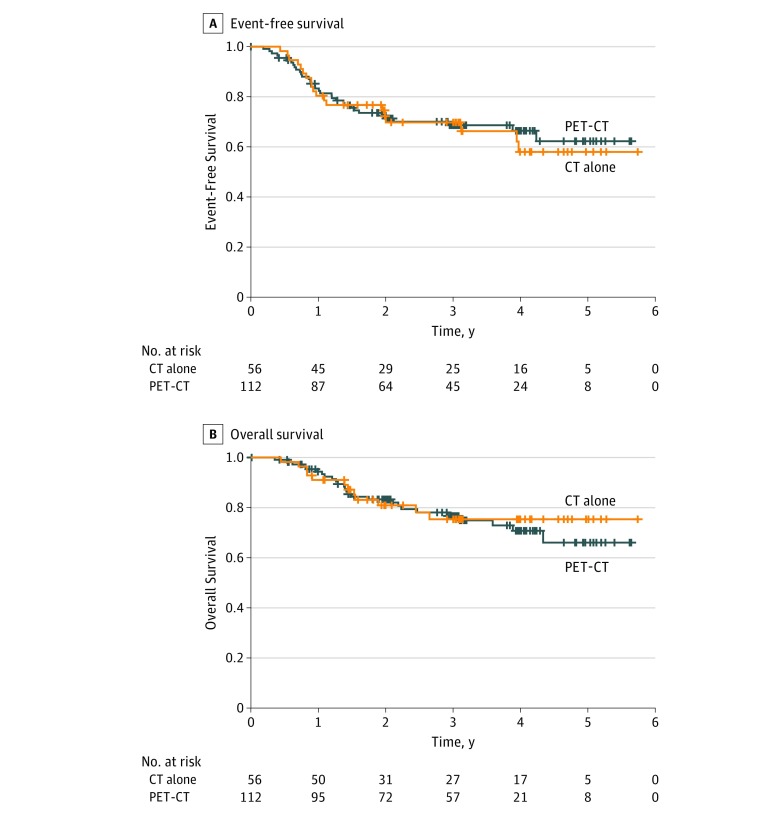
Event-Free and Overall Survival A, Event-free survival was calculated from the date of randomization to the date of objective disease recurrence, progression (positive result on a biopsy or radiologic imaging), or death due to any cause. B, overall survival was calculated from the date of randomization to the date of death due to any cause. CT indicates computed tomography; PET, positron emission tomography.

After a median (IQR) follow-up of 3.0 (0.5-5.7) years, 34 patients who underwent PET-CT imaging (30.4%) experienced recurrence or progression or died compared with 19 patients in the CT alone group (33.9%) (hazard ratio [HR] for 2-year EFS, 1.13; 95% CI, 0.64-1.99; *P* = .66) (Figure 2A). Thirty-eight patients have died, 26 in the PET-CT group (23.2%) and 12 in the CT group (21.4%) (HR, 0.97; 95% CI, 0.49-1.93; *P* = .93) (Figure 2B).

There were 103 patients in the PET-CT group who had a maximal SUV reported with a mean (SD) of 15.4 (7.4). The logarithm of SUV trended toward a negative prognostic factor; however, it was not statistically significant univariately for OS (HR, 1.58; 95% CI, 0.69-3.64; *P* = .28) or EFS (HR, 1.65; 95% CI, 0.79-3.42; *P* = .18) or after adjusting for baseline stratum (data not shown).

### Quality of Life

More than 96% of patients completed baseline QOL questionnaires, but completion rates declined to approximately 70% to 80% by 6 months and 1 year. No statistically significant difference in the global QOL score, self-perceived overall health, or overall QOL was observed between treatment groups (Table 3).

**Table 3.** Quality of Life

Events	CT of Abdomen and Pelvis (n = 56)	CT of Abdomen and Pelvis Plus Whole-Body PET-CT (n = 112)	*P* Value
Completed questionnaire, No. (%)			
Baseline	54 (96.4)	108 (96.4)	
3 mo	38 (67.9)	94 (95.7)	
6 mo	43 (76.8)	96 (85.7)	
12 mo	39 (69.6)	81 (72.3)	
18 mo	29 (51.8)	63 (56.3)	
24 mo	25 (44.6)	61 (50.0)	
Global Quality of Life Score, mean (SD)^a^			
Baseline	65.3 (20.8)	65.0 (20.6)	.75
3 mo	67.3 (21.6)	68.8 (17.1)	
6 mo	74.3 (21.0)	72.2 (19.6)	
12 mo	70.7 (22.4)	74.4 (18.6)	
18 mo	74.7 (17.4)	76.9 (20.0)	
24 mo	76.0 (19.1)	81.4 (16.9)	
How would you rate your overall health during the past week?, mean (SD)^b^			
Baseline	4.9 (1.3)	4.8 (1.3)	.96
3 mo	5.1 (1.0)	5.0 (1.3)	
6 mo	5.3 (1.2)	5.4 (1.3)	
12 mo	5.4 (1.2)	5.2 (1.2)	
18 mo	5.5 (1.2)	5.4 (1.2)	
24 mo	5.9 (0.9)	5.5 (1.2)	
How would you rate your overall quality of life during the past week?, mean (SD)^b^			
Baseline	4.9 (1.3)	5.1 (1.4)	.50
3 mo	5.2 (1.1)	5.1 (1.4)	
6 mo	5.4 (1.3)	5.5 (1.3)	
12 mo	5.6 (1.2)	5.3 (1.4)	
18 mo	5.7 (1.2)	5.6 (1.0)	
24 mo	5.8 (1.2)	5.6 (1.3)	

Abbreviations: CT, computed tomography; PET, positron emission tomography.

^a^ Scale is 0 to 100 with higher values indicating improved quality of life.

^b^ Scale is 1 to 7 with higher values indicating improved quality of life and overall health.

## Discussion

When planning our trial, we hypothesized that (1) PET-CT could detect more distant metastases than usual CT, resulting in the avoidance of curative CRT, and (2) the detection of more retroperitoneal disease would lead to more extensive radiotherapy. The detection of distant metastatic disease was relatively low, and no significant difference was detected between groups in treatment with palliative intent. The second hypothesis appeared to be supported as there was an increased rate of detection of para-aortic or common iliac adenopathy identified with PET-CT. This resulted in a 2-fold increase in radiation to extrapelvic nodes in patients in the PET-CT group, although this difference was not statistically significant, likely as a result of the small sample size due to prematurely discontinued recruitment.

There was an increased rate of detection of para-aortic or common iliac adenopathy identified with PET-CT. This change was of a similar magnitude to the increase in more extensive radiation delivered in the PET-CT group.

Survival was a secondary outcome in our trial. To date, no difference in survival has been detected between groups. The most likely explanation is insufficient power, as the number of deaths is low and longer follow-up is required. In the only other randomized trial of PET in LACC, older-technology PET (no CT) plus MRI was compared with MRI alone.^16^ No survival difference was detected between groups, but the trial was very small. Another potential explanation for lack of a survival benefit in our trial is that more extensive radiation has limited efficacy. This is supported by a study by Yap et al^17^ in which elective irradiation of occult para-aortic nodes in patients with pelvic lymph node positivity did not improve outcome. More research is needed to improve outcomes in LACC, eg, evaluating additional chemotherapy after completion of standard CRT.^18^

In our trial, although there was a trend for the SUV to be associated with worse survival, it was not statistically significant. This is in contrast to our trial of PET-CT in patients with metastatic colorectal cancer to the liver where the SUV was associated with survival.^19^

In a post hoc analysis, the presence of abnormal pelvic nodes on diagnostic CT was associated with abnormal para-aortic nodes on PET-CT.

### Limitations

The trial was stopped prematurely as a result of slow rate of recruitment. The reason is unclear. It is unlikely that patients were able to have a PET-CT outside of the trial, as the government-funded health care system would not cover the cost. Another possible explanation is an overly optimistic estimate of the number of patients with the disease of interest. The failure to detect a statistically significant difference for the primary outcome was likely a result of low power from the small sample size.

The trial design assumed that the investigators would follow the recommended treatments as described in the study protocol. The final radiation prescription was left to the treating radiation oncologist. Unfortunately, in the PET-CT group, a substantial number of patients with normal nodes received more extensive radiation, which was not expected based on the study protocol. The net effect might be to overestimate the effect of staging with PET-CT. The reasons for nonadherence to the protocol are unclear. Perhaps it reflected the beliefs of radiation oncologists related to the use of more extensive radiation for microscopic nodal disease too small to be detected by PET-CT.

The results of our trial are generalizable to patients presenting with LACC defined by clinical staging using the FIGO criteria.^10^ Most would not be candidates for primary surgery. In many jurisdictions, these patients would be offered only CRT. Radical hysterectomy is used for FIGO stage IB1 disease if there are no contraindications to surgery. Only 5% of patients in our trial had this stage of disease.

## Conclusions

The medical costs of caring for patients with cancer are high and are expected to increase in the future because of new treatments, new technology, and aging of the population.^20^ The high costs of imaging in patients with cancer are a component of this increase.^20,21^ An Institute of Medicine report from 2009 prioritized comparative effectiveness research in the area of imaging technologies in diagnosing and staging patients with cancer, including the use of PET-CT, CT, and MRI.^21,22,23^ Our clinical trial fits with the aims of comparative effectiveness research.

In the United States, PET-CT has been adopted for staging of LACC based on small studies conducted more than a decade ago that evaluated the accuracy of the test in detecting lymph nodes and not its utility.^24^ In other countries, PET-CT for the staging of LACC has not been widely adopted. The 2017 National Comprehensive Cancer Network guidelines for cervical cancer recommend PET-CT (preferred) or chest, abdomen, and pelvic CT for the initial workup of stage II to IV cervical cancer.^25^ This recommendation is based on level 2A evidence, which is defined as “based on lower level evidence there is uniform consensus that the recommendation is appropriate.”^25^ Our trial is the only randomized clinical trial that we know of in the contemporary era that actually addresses the utility of PET-CT for LACC.^26^ Although our trial was underpowered, it is reasonable to consider the results within the context of the National Comprehensive Cancer Network recommendations that are based on observational data. Our results provide higher-quality evidence to support current practice.^27^ Finally, a prudent and efficient approach might be to consider PET-CT only for patients with abnormal pelvic nodes on CT. Such a policy is currently funded by the Ontario Ministry of Health.

## Notes

Supplement 1. **Trial Protocol**

Supplement 2. **eTable 1.** Radiotherapy Guidelines **eAppendix.** Sample Size Calculation **eTable 2.** Lymph Nodes, PET-CT, and Treatment Delivered **eTable 3.** Lymph Nodes, CT, and Treatment Delivered
