# Supplementary material for: Effect of Positron Emission Tomography Imaging in Women With Locally Advanced Cervical Cancer: A Randomized Clinical Trial
Source: JAMA Netw Open. 2018 Sep 14;1(5):e182081. doi: 10.1001/jamanetworkopen.2018.2081 (PMC6324512; doi:10.1001/jamanetworkopen.2018.2081)
Supplement: Supplement 1. — Trial Protocol [file jamanetwopen-1-e182081-s001.pdf]

CONFIDENTIAL

CLINICAL TRIAL PROTOCOL

The Impact of Positron Emission Tomography (PET) Imaging in Women with Locally Advanced Cervical Cancer

PET LACE

Protocol Number: OCOG-2009-PETLACE

Protocol Version: Version 5.0

Protocol Date: April, 01, 2011

Sponsor: Ontario Clinical Oncology Group

The material contained in this document is confidential information belonging to the Ontario Clinical Oncology Group (OCOG), Department of Oncology, McMaster University, Hamilton Health Sciences, Hamilton, ON, Canada and the Study Steering Committee. Except as may be otherwise agreed to in writing, by accepting or reviewing these materials, you agree to hold such information in confidence and not to disclose it to others (except where required by applicable law) nor use it for unauthorized purposes. In the event of actual or suspected breach of this obligation, OCOG should be notified.

PRINCIPAL INVESTIGATOR(S)

Dr. Anthony Fyles
UHN- Princess Margaret Hospital
610 University Avenue,
Toronto, ON M5G 2M9
Tel: (416)-946-6522
Fax: (416)-946-2111
Email: anthony.fyles@rmp.uhn.on.ca

____

Signature

____

Date

Dr. Laurie Elit
Juravinski Cancer Centre
699 Concession Street,
Hamilton, ON L8V 5C2
Tel: (905)-389-5688
Fax: (905)-575-6343
Email: laurie.elit@jcc.hhsc.ca

____

Signature

____

Date

Dr. Douglas Coyle
University of Ottawa
Epidemiology & Community Medicine
451 Smyth Road,
Ottawa, ON K1H 8M5

Tel: (613)-562-5800 ext. 8690
Fax: (613)-562-5465
Email: dcoyle@uottawa.ca

____

Signature

____

Date

SPONSOR
Ontario Clinical Oncology Group (OCOG)

Dr. Mark Levine
Director, Ontario Clinical Oncology Group
McMaster University, Faculty of Health Sciences, Department of Oncology
Henderson Research Centre
711 Concession Street
Hamilton, ON L8V 1C3

____

Signature

____

Date

Qualified Investigator Acknowledgement

This acknowledgement serves to document the agreement of the Qualified Investigator at each participating clinical centre to conduct this clinical trial in accordance with the dated version of the protocol as specified. The dated version of the protocol is agreed to by the Sponsor, the Ontario Clinical Oncology Group (OCOG) and the clinical trial Principal Investigator(s), and has been approved by Health Canada, if applicable.

**PROTOCOL**	**Title:** **The Impact of Positron Emission Tomography (PET) Imaging in Women with Locally Advanced Cervical Cancer (PETLACE)**<br><br>**Number: OCOG-2009-PETLACE**
**VERSION**	Version 5.0
**DATE**	April, 01, 2011

I have read this clinical trial protocol and by signing this form, agree to the following:

(i) To maintain confidentiality. Understanding that this protocol and any supplemental information that may be added is confidential information and is the property of OCOG.

(ii) To conduct this clinical trial in accordance with this protocol, applicable national and local regulations related to the conduct of research studies involving human subjects; ICH Good Clinical Practice guidelines.

(iii) To ensure that personnel designated to perform study related procedures are under my supervision and that medical care and decisions made in respect of this study are my responsibility. To maintain and provide to OCOG documentation of the delegation of study related procedures.

QUALIFIED INVESTIGATOR (one per Clinical Centre)

Dr.
Clinical Centre
Address 1
Address 2
City, PROVINCE Postal Code

____

Investigator (print name)

____

Signature Date

STUDY SCHEMA

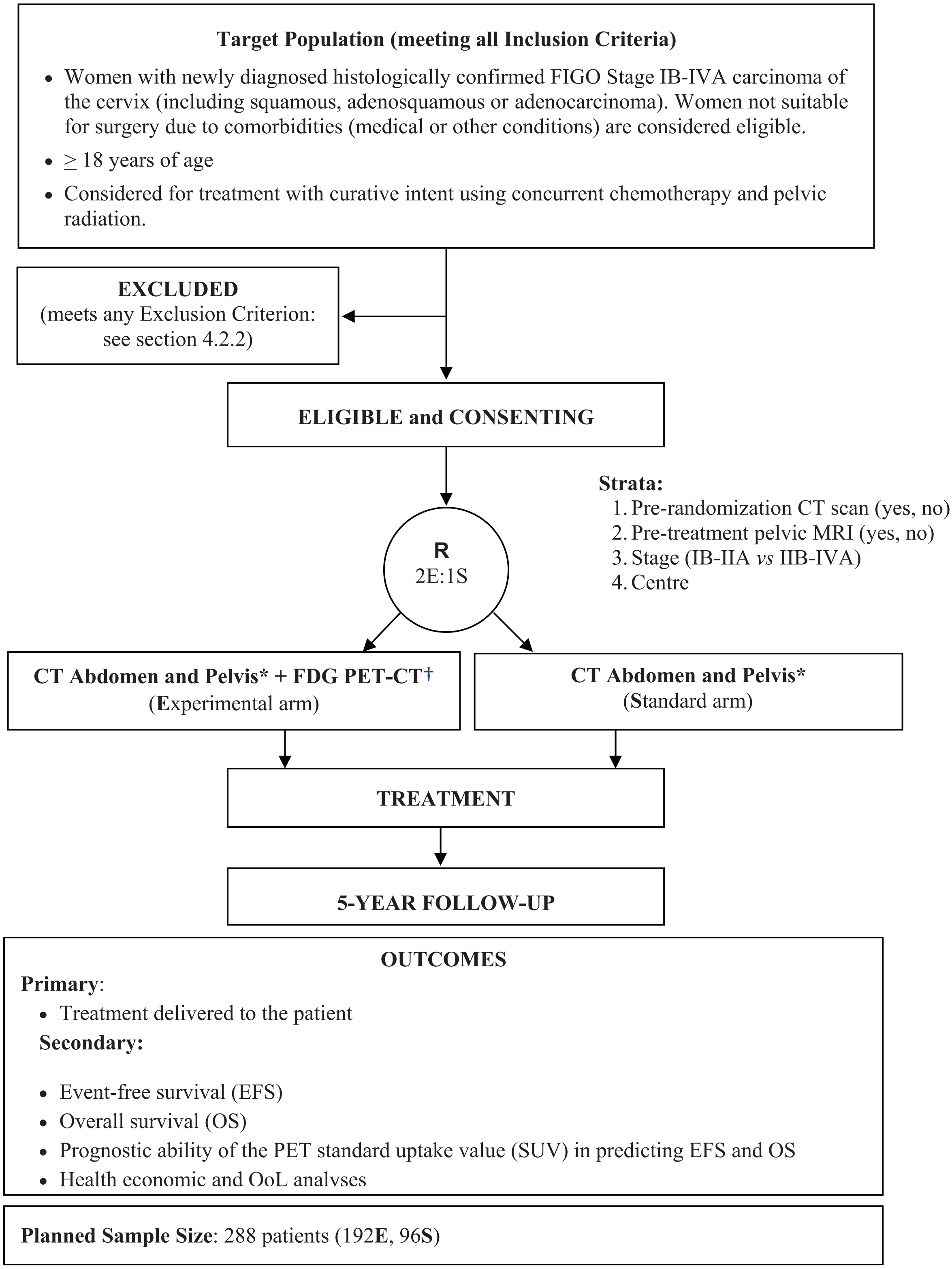

* Usual strategy is contrast-enhanced CT of abdomen and pelvis

† If contrast-enhanced CT of abdomen and pelvis has been done prior to randomization, then a low resolution CT is done with the PET

LIST OF ABBREVIATIONS USED IN THIS PROTOCOL

ACRIN	American College of Radiology Imaging Network
AE	Adverse Event
BGTD	Biologics and Genetic Therapies Directorate
CAC	Central Adjudication Committee
CIHI	Canadian Institute of Health Information
CMC	Coordinating and Methods Centre
CRF	Case Report Form
CRT	Conventional Radiotherapy
CT	Computerized Tomography
DRRs	Digitally Reconstructed Radiographs
DSMB	Data Safety Monitoring Board
ECOG	Eastern Cooperative Oncology Group
EDC	Electronic Data Capture
EFRT	Extended Field Radiation Therapy
EFS	Event Free Survival
EORTC	European Organization for Research and Treatment of Cancer
^{18}FDG PET	18F-fluorodeoxyglucose Positron Emission Tomography
FIGO	International Federation of Gynecology and Obstetrics
GCP	Good Clinical Practice
HDR	High Dose Rate
IEC	Independent Ethics Committee
IMRT	Intensity Modulated Radiation Therapy
IRIS	Interactive Registration/RandomIzation System
LDR	Low Dose Rate
MLC	Multi-leaf Collimator
MOHLTC	Ministry of Health and Long Term Care
MRI	Magnetic Resonance Imaging
NCI-CTCAE	National Cancer Institute Common Terminology Criteria for Adverse Events
OCOG	Ontario Clinical Oncology Group
ORCCID	Online Remote Collection of Clinical Information and Data
OS	Overall Survival
PDR	Pulse Dose Rate
PMH	Princess Margaret Hospital
QA	Quality Assurance
QALY	Quality Adjusted Life Years
QI	Qualified Investigator
QoL	Quality of Life
RCT	Randomized Controlled Trial
REB/IRB	Research Ethics Board/Institutional Review Board
RT	Radiation Therapy
RTOG	Radiation Therapy Oncology Group
SAE	Serious Adverse Event
SUV	Standardized Uptake Value

TABLE OF CONTENTS

STUDY SCHEMA 5

LIST OF ABBREVIATIONS USED IN THIS PROTOCOL 6

1. BACKROUND INFORMATION AND RATIONALE 9
1.1. INTRODUCTION 9
1.2. TUMOUR STAGE 9
1.3. TREATMENT 9
1.4. POSITRON EMISSION TOMOGRAPHY (PET) 11
1.5. PET IMAGING FOR CERVICAL CANCER STAGING 11
1.6. UPSTAGING AND TREATMENT 15
1.7. WORK OF INVESTIGATORS 16
1.8. STUDY RATIONALE 16
2. STUDY OBJECTIVES 17
2.1. GENERAL OBJECTIVE 17
2.2. PRIMARY OBJECTIVE 17
2.3. SECONDARY OBJECTIVES 17
3. STUDY DESIGN 17
4. STUDY POPULATION 18
4.1. SCREENING FOR POTENTIALLY ELIGIBLE PATIENTS 18
4.2. ELIGIBILITY CRITERIA 18
4.2.1. INCLUSION CRITERIA 18
4.2.2. EXCLUSION CRITERIA 18
5. PRE-TREATMENT EVALUATION 19
6. RANDOMIZATION 20
6.1. STRATIFICATION 20
6.2. RANDOMIZATION PROCEDURE 20
7. STUDY INTERVENTION 20
7.1. CT Abdomen and Pelvis + WHOLE-BODY PET-CT GROUP (EXPERIMENTAL ARM) 20
7.2. CT ABDOMEN AND PELVIS GROUP (STANDARD ARM) 21
7.3. POST-IMAGING DIAGNOSTIC CONFIRMATION 21
7.4. QUALITY ASSURANCE 22
8. STUDY TREATMENT 22
8.1. STANDARD RADIATION THERAPY FOR LOCALLY ADVANCED CERVICAL CANCER 22
8.1.1. PELVIC RADIATION 22
8.1.1.1. External Irradiation 22
8.1.1.2 Pelvic Boost 24
8.1.2. INTRACAVITARY BRACHYTHERAPY 24
8.2. STANDARD CHEMOTHERAPY FOR LOCALLY ADVANCED CERVICAL CANCER 25
9. CONCOMITANT MEDICATION 25
9.1. PROHIBITED CONCOMITANT MEDICATION AND THERAPY 25
10. EVALUATION DURING AND AFTER TREATMENT 25
10.1. TREATMENT PERIOD ASSESSMENT 25
10.2. FOLLOW-UP ASSESSMENT 25
11. ADVERSE EVENTS 26
11.1. ADVERSE EVENT DEFINITIONS 26
11.2. ATTRIBUTION DEFINITIONS 27
11.3. ADVERSE EVENT REPORTING CRITERIA 27
11.4. ADVERSE EVENT REPORTING PERIOD 28
11.5. SERIOUS ADVERSE EVENT REPORTING TO OCOG 28
11.6. SERIOUS ADVERSE EVENT REPORTING TO HEALTH CANADA 29
11.7. REPORTING SAEs TO LOCAL RESEARCH ETHICS BOARDS 29
12. STUDY OUTCOMES 29
12.1. PRIMARY OUTCOME 29

12.2. Secondary Outcomes ... 30
13. STATISTICAL CONSIDERATIONS ... 30
13.1. Statistical Analysis ... 30
13.2. Sample Size and Feasibility ... 31
13.3. Analysis Sets ... 32
13.4. Health Related Quality of Life Data ... 32
13.5. Health Economic Data ... 32
13.6. Planned Interim Analysis ... 33
14. CENTRAL ADJUDICATION ... 33
15. STUDY SIGNIFICANCE ... 33
16. ETHICAL AND REGULATORY STANDARDS ... 33
16.1. Informed Consent ... 34
16.2. Research Ethics Board (REB) or Institutional Review Board (IRB) ... 34
17. RESPONSIBILITIES OF THE INVESTIGATOR ... 35
17.1. Source Document Requirements ... 35
17.2. Case Report Forms (CRFs) ... 35
18. CONFIDENTIALITY ... 36
19. CLINICAL TRIAL PROTOCOL AMENDMENTS ... 36
20. STUDY ORGANIZATION ... 36
20.1. Steering Committee ... 36
20.2. Data Safety Monitoring Board ... 37
20.3. Study Coordination ... 37
20.4. Central Adjudication Committee (CAC) ... 37
21. SCIENTIFIC REPORTING AND PUBLICATION ... 38
22. REFERENCES ... 39

APPENDIX I: CARCINOMA OF THE CERVIX: FIGO NOMENCLATURE ... 42

APPENDIX II: ECOG PERFORMANCE STATUS* ... 44

APPENDIX III: SCHEDULE OF STUDY ASSESSMENTS AND EVALUATIONS ... 45

APPENDIX IV: QUALITY OF LIFE INSTRUMENTS ... 46

APPENDIX V: HEALTH UTILITY INSTRUMENT ... 51

APPENDIX VI: ^{18}FDG PET-CT + CONTRAST-ENHANCED CT ABDOMEN AND PELVIS SCANNING PROCEDURE ... 53

APPENDIX VII: ^{18}FDG PET IMAGE INTERPRETATION ... 57

APPENDIX VIII: AMINOGLYCOSIDE ANTIBIOTICS ... 58

APPENDIX IX: ECONOMIC ANALYSIS ... 59

1. BACKROUND INFORMATION AND RATIONALE

1.1. Introduction

Cervical cancer is the second most common cause of cancer deaths worldwide[1]. In Canada, it is estimated that in 2009 there will be 1,300 new cases of cervical cancer and that 380 women will die of this disease. The corresponding 2009 data for Ontario is 500 new cases and 140 deaths[2]. In Canada, cervical cancer screening with the Pap test allows for the diagnosis and curative treatment of precancerous lesions of the cervix and early cervical cancers[3,4]. Symptoms of cervical cancer include vaginal bleeding and discharge. Unfortunately these are often associated with more advanced disease.

1.2. Tumour Stage

Carcinoma of the cervix is staged clinically at presentation and the stage reflects the burden of tumour (***Appendix I***) [5,6]. The stage also influences the treatment of the disease and its prognosis. Cervical cancer has an orderly pathway of spread initially involving the cervix, then the upper vagina and para-cervical tissue. During this process the pelvic nodes and eventually the para-aortic nodes become involved. Metastases to the peritoneum, liver and bone are late manifestations of the disease. Stage is the strongest predictor of outcome with early stage disease being associated with the highest cure rates and locally advanced and metastatic disease with the lowest cure rates (Table 1).

Table 1: FIGO Stage and Outcome

Stage	Number of patients	5-yr overall survival
IA1	860	98.7%
IA2	227	95.9%
IB1	2530	88.0%
IB2	950	78.8%
IIA	881	68.8%
IIB	2375	64.7%
IIIA	160	45.3%
IIIB	1949	47.6%
IVA	245	24.1%
IVB	189	17.2%

1.3. Treatment

Early stage cervical cancer (Stages IA-IB1) is treated surgically either with a limited procedure e.g. a cone biopsy of the cervix or a simple hysterectomy, or a radical hysterectomy and pelvic node dissection for cancer up to 2cm in diameter. Chemotherapy plus radiation therapy (RT) is the standard of care for disease larger than this (Stages IB2-IVA [also referred to as locally advanced disease]) or for women who are not surgical

candidates as a result of co-morbid diseases which would preclude a long operation. Eight randomized controlled trials in women with locally advanced cervix cancer have shown a statistically significant improvement in survival with cisplatin-based chemotherapy plus pelvic RT and intracavitary boost compared with RT alone (relative risk of death 0.74 95% CI 0.64-0.86).[7] Combined modality therapy with cisplatin-based chemotherapy and RT has become the standard of care in Canada[8]. The usual radiation regimen consists of external pelvic RT plus intracavitary boost using brachytherapy. This treats the disease in the cervix and surrounding tissues, as well as the first-echelon pelvic lymph nodes (i.e., internal and external iliac and pre-sacral nodes).

Women die from cervical cancer for two reasons, either the disease cannot be controlled in the irradiated field or disease outside the radiation field at the time of diagnosis is not recognized. This latter cause of treatment failure can be addressed by surgically staging pelvic and para-aortic nodes either by laparotomy or more recently by laparoscopy, at the expense of potential delay in definitive treatment. Of particular importance is presence of cancer in the para-aortic nodes as these are not treated during pelvic RT. The risk of nodal involvement is related to stage: 7% of women with Stage IB disease have positive para-aortic nodes[9]; whereas in one series, 21% of women with Stage IIB, 31% of Stage III, and 13% of patients with Stage IVA disease had positive para-aortic nodes, respectively[10]. Data from other studies with similar findings is shown in Table 2[11].

Table 2: Incidence of Para-aortic Lymph Node Metastases by Stage

Study	Stage II			Stage III		
	Explored	Positive	Incidence %	Explored	Positive	Incidence %
Nelson 1977	63	9	14.3	39	15	38.5
Delgado 1977	18	8	44.4	13	5	38.5
Piver and Barlow 1977	46	6	13.0	49	18	36.7
Sudarsanam 1978	43	7	16.3	19	3	15.8
Buchsbaum 1979	19	1	5.3	104	34	32.7
Hughes 1980	80	14	17.5	96	23	24.0
Ballon 1981	48	9	18.8	24	4	16.7
Welander 1981	63	13	20.6	38	10	26.3
Berman 1984	265	43	16.2	180	45	25.0
Potish 1985	47	5	10.6	11	4	36.4
La Polla 1986	47	6	12.8	38	14	36.8
**Total**	**739**	**121**	**16.4**	**611**	**175**	**28.6**

Only 6% of patients with locally advanced cervix cancer undergo staging laparotomy because of its high rate of morbidity especially when followed by RT and the potential for

delaying definitive treatment. The usual practice prior to therapy is to stage the cervical cancer with non-invasive imaging tests, e.g. CT of the abdomen and pelvis and sometimes MRI to guide treatment (MRI is done to image the cervix and para-cervical tissues). However, the accuracy of these imaging tests for staging lymph nodes is limited. There have been reports of laparoscopic sentinel node biopsy to detect nodal disease; however, this is still exploratory and not the standard of care across Canada. An accurate non-invasive functional imaging method for nodal staging could add important information to anatomical staging with CT and MRI and would be valuable in optimizing RT.

1.4. Positron Emission Tomography (PET)

Traditional radiological imaging (e.g. CT, MRI) is based on structural information and defines disease states based on gross anatomical changes whereas PET imaging is based on biochemical processes that often precede any gross anatomic distortion. PET imaging is now used primarily in oncologic imaging due to the successful application of ^{18}F-fluorodeoxyglucose (FDG). ^{18}FDG is preferentially taken up in tumour tissue because increased glycolysis is associated with malignancy compared to most normal tissue[12-16]. Imaging by PET is based on the detection of 511 KeV annihilation photons that is the result of a positron, in this case emitted from ^{18}F, colliding with an electron. The collected data is then processed into the final image of FDG localization. The PET image does not provide accurate anatomical information aside from those areas of normal physiological uptake (such as the heart, kidneys and bladder) and soft tissue uptake (e.g. muscle) that can provide an outline of the imaged body. Therefore, advances in imaging technology have combined PET and computed tomography (PET-CT) to provide both functional and anatomical information simultaneously, thus improving its accuracy. [17-22]

1.5. PET Imaging for Cervical Cancer Staging

There have been multiple studies where patients with carcinoma of the cervix had imaging tests (including PET) and then underwent surgical staging of the pelvic and abdominal lymph nodes. Havrilesky and colleagues conducted a systematic review of prospective studies in which ^{18}FDG PET was used and patients also had staging aortic node lymphadenectomy[23] (Table 3).

Table 3: Accuracy of Detecting Para-aortic or Pelvic Nodes by Various Imaging Modalities

Author	N	PET		MRI		CT	
**(a) Accuracy for Detection of Para-aortic Nodes**							
	**N**	**Sensitivity**	**Specificity**	**Sensitivity**	**Specificity**	**Sensitivity**	**Specificity**
Reinhardt	12	1.0	1.0	0.67	1.0		
Rose	32	0.75	0.92			All had prior negative CT	
Lin	50	0.86	0.94			All had prior negative CT	
Yeh	42	0.83	0.97	All had negative MRI			
**Pooled**		**0.84**	**0.95**				
**(b) Accuracy for Detection of Pelvic Nodes**							
Reinhardt	35	0.91	1.0	0.73	0.83		
Sugarawa	13	1.0	1.0			0.50	1.0
Rose	32	1.0	1.0			0.45	
Wright		0.52	0.90				
Belhocine	22	0.56	0.98	0.72	0.97		
**Pooled**		**0.79**	**0.99**				

The pooled sensitivity for ^{18}FDG PET for detection of para-aortic nodes was 0.84 and the pooled specificity was 0.95. There was insufficient information on conventional imaging with MR and CT. These authors also identified four studies on the accuracy of PET for pelvic nodes. Two studies used lymphadenectomy as the gold standard and two used clinical follow-up. The pooled sensitivity was 0.79 and pooled specificity was 0.99. Two studies each had data on CT and MRI. Although the numbers are small, there is a suggestion that the accuracy of PET is better than that for CT or MRI. More recent studies have supported the notion that PET is more accurate than CT or MRI in identifying disease.[24-26]

There have been other case series that reported on staging of cervix cancer with PET imaging[27-29]. In summary, the available evidence suggests that PET is more accurate than conventional imaging. However, the number of patients studied is relatively small, the studies are old (2005 or earlier) and uncontrolled and none of the studies used modern PET-CT.

Thus, PET-CT imaging can upstage patients, with the potential to improve treatment for patients with more extensive disease. An example of this might be a patient with normal-sized pelvic nodes on CT scan who would be treated more effectively with para-aortic RT if

PET-CT demonstrated positive nodes. Conversely, if PET-CT detected metastatic disease outside the pelvis and para-aortic nodes not found by conventional imaging (e.g. in one study 8% of patients had supraclavicular nodal metastases detected by PET[29]), these patients would be spared chemo-RT and receive palliative care.

There have been few high quality studies that have shown that staging with PET has a major clinical impact. Review of 447 papers from 2000 to 2008 on PET and cervix cancer found only 11 with information on the clinical impact of PET imaging, and none of very high quality (as determined by the grading scheme of the National Health Service Health Technology Assessments, Table 4). In the only randomized trial reported by Tsai, 129 women with positive pelvic and negative para-aortic nodes on MRI were allocated to PET or no further imaging. PET imaging detected extra-pelvic disease in 7 of 66 patients (11%), with 6 para-aortic nodes and one omental node. The six patients with para-aortic nodes received extended radiation to the para-aortic region, but there were no differences in 4-year overall survival (79% vs. 85% PET vs. no PET, p = 0.65), disease-free survival (75 % vs. 77%), and distant metastasis-free survival (82% vs. 78%). In the non-randomized study by Yen, PET imaging was performed in 135 women with cervical cancer, 35% with locally advanced and 65% with recurrent disease[30]. Twenty-eight percent of these women had their RT changed based on the PET, but the results were not reported for the locally advanced patients alone. In the Bjurberg study RT was altered in 23.5% of 17 patients with FIGO Stage IB-IVB disease based on PET[31]. Narayan would have modified management based on PET in 14 of 27 (52%) of women. Thus management change ranged from 10 to 52%, depending on the patient group, baseline staging and definition of change in management (Table 4). This limited data suggests that PET may have an important clinical impact but the lack of large prospective randomized trials has hampered its development as a staging modality. Thus the role for PET and PET-CT in upstaging patients leading to changes in treatment delivered and possibly outcome are unclear. The only randomized trial was limited to women with positive pelvic nodes and was under-powered to detect small but meaningful differences in disease free survival and overall survival.

Table 4: PET Cervix Studies Describing Change in Management

Author Date	Design	Change in treatment
Tsai<br>In press	PET vs. no PET<br>(MRI pos pelvic nodes)	10.6% (7/66)<br>n= 6: Pelvic toEFRT
Dolezelova<br>2008	PET + CT	17.6% (9/51)<br>RT field change
Chao<br>2008	CT/MRI then PET or PET-CT<br>47 pts +ve PALN/Ing.LN/SCF LN for "curative Mx"	44.7% (21/47) +ve clinical impact<br>6.4% (3/47) –ve clinical impact
Yildrim<br>2008	CT then PET-CT then PALN dissection pre-Tx	25% (4/16) would have had management changed based on PET<br>Treatment actually based on surgical staging
Bjurberg<br>2007	CT +/- MRI pelvis and PET-CT	23.5% (4/17) pts
Amit<br>2006	PET-CT	13% (10/75)<br>5 EFRT, 5 surgery to RT
Ma<br>2003	CT/MRI then dual phase PET	10.3% (4/39) pts had RT field changed, but 16/39 had PALN detected
Yen<br>2003	CT/MRI then dual phase PET	21.5% (29/135) pts<br>23: RT field +/or dose changed<br>6: Intent changed to palliation
Belhocine<br>2002	MRI +/-CT then PET in 2 gps of pts (22 relevant)	18.2% (4/22) pts
Narayan<br>2001	MRI + CT then PET then PALN dissection	51.9% (14/27) pts would have had modified management (i.e. add pelvic boost or EFRT)
Grigsby<br>2001	CT + PET	13.9% (14/101) pts "could have had EFRT" due to PALN detected only on PET

Another potential application of PET is to provide prognostic information on the tumour by providing data for calculation of the standardized uptake value (SUV). The SUV has been prognostic in other tumours (i.e. lung cancer). There is some very preliminary data in cervical cancer[32-35], but it is not clear that SUV is independent of traditional clinical prognostic factors.

1.6. Upstaging and Treatment

When the pelvic nodes and para-aortic nodes are normal on imaging, patients receive pelvic RT (45-50 Gy by external beam to the level of L5/S1 plus brachytherapy) and weekly cisplatin-based chemotherapy. The detection of tumour in the common iliac or para-aortic nodes by an imaging test leads to a change in the RT volume of the combined modality treatment (suggested treatments indicated in Table 5). Patients receive pelvic RT and para-aortic RT (40-45 Gy) to include para-aortic nodes to the T12/L1 level. This RT is also referred to as extended field RT (EFRT). The addition of EFRT increases the side effects of treatment e.g. 3% major toxicity to the small bowel[36]. If imaging detects disease in the common iliac nodes, the pelvic RT fields are altered to include the next echelon lower para-aortic nodes up to L1/L2. The detection of tumour in the pelvic nodes may also lead to a change in treatment with an increase in the pelvic RT field to the L4 level. Finally, if imaging detects distant disease e.g. supraclavicular or mediastinal nodes, bone, liver, or lung, the patient is no longer a candidate for combined modality chemo-RT and can receive symptom management and/or palliative care. In some cases, PET-CT may downstage nodes or metastatic sites thought to be involved on the basis of CT, resulting in less extensive treatment.

Table 5: Recommended Changes in Therapy

<table>
<tr><th>Imaging</th><th colspan="2">Recommended treatment</th></tr>
<tr><td>Disease in Para-aortic nodes</td><td rowspan="3">EFRT</td><td>Extend radiation field to include Para-aortic nodes to T12/L1</td></tr>
<tr><td>Disease identified in pelvic nodes or presacral nodes</td><td>Extend superior pelvic margin to include common iliac nodes to L4 and/or include whole sacrum</td></tr>
<tr><td>Disease identified in common iliac nodes</td><td>Extend superior pelvic field margin to include low Para-aortic nodes i.e., L1/2</td></tr>
<tr><td>Extensive disease identified in primary cervix tumor or pelvic nodes</td><td colspan="2">Modify pelvic radiation fields without EFRT</td></tr>
<tr><td>More extensive cervical disease e.g. uterine extension</td><td colspan="2">Change brachytherapy volume or dose</td></tr>
<tr><td>Supraclavicular nodes, mediastinal nodes, bony mets, other solid organ disease</td><td colspan="2">Palliative care only ie., chemo alone or palliative radiation therapy, clinical trials or supportive care</td></tr>
</table>

1.7. Work of Investigators

The Ontario Clinical Oncology Group (OCOG) has led the field evaluation program for PET in oncology in Ontario. OCOG has worked with a network of investigators to design, implement and conduct six clinical trials evaluating ^{18}FDG PET for the following clinical indications; staging of early lung cancer patients being considered for surgery, staging of patients with locally advanced lung cancer and using ^{18}FDG PET to plan radiation, detection of neck metastases post-radiation in head & neck cancer, staging of the axilla in women with early breast cancer who undergo sentinel node biopsy, staging of colorectal cancer metastatic to the liver prior to surgery, and diagnosing recurrence of tumour in situations where conventional imaging has been inconclusive. To-date, 1800 patients have been entered in these trials.

Three of these studies are randomized trials and three are prospective cohort studies. Three have been completed and presented at international meetings[37-39]. As part of the evaluation project, OCOG organized a quality assurance process that included: reading of PET scans by nuclear medicine physicians, standardizing the dose of FDG used for PET imaging across the province, and calibration of the five PET scanners to the same standards.

The dedicated group of radiation, medical and gynecologic oncologists participating in this study has extensive experience in enrolling patients into cervical cancer trials through co-operative trials groups. In addition, there is an experienced team of radiation oncologists, radiologists and nuclear medicine physicians who have participated in the six OCOG PET studies.

1.8. Study Rationale

This is an opportune time to perform a trial in locally advanced cervical cancer because there is considerable debate worldwide regarding the utility of PET for staging cervical cancer. Although there are studies on the accuracy (sensitivity and specificity) of PET in cervical cancer, there is only one small prospective randomized study on how PET information affects treatment decisions and outcomes. As a result, PET may be underutilized for staging cervix cancer[40].

The costs associated with health care are increasing and PET is an expensive imaging modality. Given that resources for health care are not unlimited, there needs to be high quality evidence of an intervention such as PET's efficacy. To this end, the Ministry of Health and Long Term Care (MOHLTC) has been sponsoring a field evaluation consisting of clinical trials to generate the evidence. There are a core number of PET-CT units available in Ontario, and because of past work in PET and oncology, the clinical protocols are standardized. With the effort of OCOG and the Ontario MOHLTC, there is a system in place to complete this work.

2. STUDY OBJECTIVES

2.1. General Objective

To improve the clinical management and outcome of patients with locally advanced cervical cancer by using ^{18}FDG PET-CT imaging.

2.2. Primary Objective

In women with FIGO Stages IB-IVA carcinoma of the cervix who are candidates for chemo-RT, to compare the treatments delivered between patients having whole body ^{18}FDG PET-CT in addition to CT of the abdomen and pelvis versus patients having a CT of the abdomen and pelvis alone. The treatment delivered will be classified as standard pelvic chemo-radiotherapy of curative intent; more extensive chemo-radiotherapy of curative intent such as EFRT; or therapy with palliative or non-curative intent (i.e. which may include symptom management, radiation, chemotherapy or combined modality therapy, used to maximize disease and symptom control).

2.3. Secondary Objectives

In women with FIGO Stages IB-IVA carcinoma of the cervix who are candidates for chemo-RT:

1. To compare the event-free survival (EFS) of patients who undergo pre-treatment imaging using ^{18}FDG PET-CT in addition to CT of the abdomen and pelvis with the EFS of patients who undergo pre-treatment CT of the abdomen and pelvis alone.
2. To compare the overall survival (OS) of patients who undergo pre-treatment imaging using ^{18}FDG PET-CT in addition to CT of the abdomen and pelvis with the OS of patients who undergo pre-treatment CT of the abdomen and pelvis alone.
3. To investigate whether the PET standardized uptake value (SUV) of cervical cancer is prognostic for EFS and OS.
4. To conduct economic and quality of life (QoL) analyses of the strategy of pre-treatment imaging using ^{18}FDG PET-CT in addition to CT of the abdomen and pelvis compared with a strategy of pre-treatment CT of the abdomen and pelvis alone.

3. STUDY DESIGN

A multicentre randomized controlled trial to determine the impact of pre-treatment staging with whole-body ^{18}FDG PET-CT in addition to CT of the abdomen and pelvis compared to CT of the abdomen and pelvis alone in patients with potentially curable cervical cancer being assessed for chemo-RT. (Refer to Study Schema).

After initial assessment, eligible consenting patients will be randomized to either pre-treatment whole-body ^{18}FDG PET-CT in addition to CT of the abdomen and pelvis or CT of the abdomen and pelvis alone. Patients with disease confined to the pelvis will be treated with standard chemo-RT. If there is suspicion of metastatic disease, investigators will be encouraged to perform additional investigations to confirm the presence of the disease histologically, if possible (although this is not mandatory). If investigation or biopsy of

suspicious lesion(s) is inconclusive or not performed, the patient will receive treatment at the discretion of the radiation oncologist. If the patient has metastatic disease outside of potentially radiated areas, she will be offered palliative therapy either by chemotherapy alone, or palliative RT or symptomatic management. The primary outcome is treatment delivered to the patient. Patients will be followed for approximately 5 years.

4. STUDY POPULATION

4.1. Screening for Potentially Eligible Patients

Written informed consent will be obtained from all potentially eligible patients prior to commencing any study related screening or treatment procedures. Until the patient has been completely informed of the clinical trial, has freely consented to take part in the study and has signed and dated an informed consent form that has received documented approval by a licensed Research Ethics Board (REB), no study related procedures, including those required for screening can be performed.

4.2. Eligibility Criteria

Eligibility status must be confirmed by the local investigator prior to randomization. It is important that no exceptions be made to the eligibility criteria. Questions related to eligibility requirements and/or specific criteria must be discussed with OCOG prior to randomization.

4.2.1. Inclusion Criteria

For inclusion in this study, patients must fulfill all of the following criteria:

1. Women with newly diagnosed histologically confirmed FIGO Stage IB-IVA carcinoma of the cervix (see ***Appendix I***), including squamous, adenosquamous or adenocarcinoma. Women not suitable for surgery due to comorbidities (medical or other conditions) are also considered eligible;

2. ≥ 18 years of age;

3. Being considered for treatment with curative intent using concurrent chemotherapy and pelvic RT.

4.2.2. Exclusion Criteria

Patients who satisfy any of the following exclusion criteria are NOT eligible for this study. These criteria will apply only to those patients who first satisfy the inclusion criteria:

1. ECOG performance status > 2 (see ***Appendix II***)
2. Other cervical cancer tumour types (e.g. neuroendocrine, serous)
3. Carcinoma of the cervical stump
4. Prior hysterectomy
5. Patients who, at the time of the initial evaluation, have already undergone a whole body PET-CT within the last 6 months

6. Contraindications to ^{18}FDG PET-CT
7. Inability to lie supine for imaging with PET-CT
8. Contraindication to radiotherapy (i.e., significant Crohn's disease)
9. Contraindication to cisplatin chemotherapy (i.e., non-reversible renal failure)
10. Inadequate bone marrow function: ANC <1.5 x 10^9, platelets <100 x 10^9
11. Inadequate renal function: Creatinine ≥150 micromol/L
12. Inadequate hepatic function: Bilirubin >1.5 x ULN and SGOT and Alkaline Phosphatase >3 x ULN
13. History of another invasive malignancy within the previous 5 years with the exception of non-melanoma skin cancer
14. Other medical conditions that may preclude chemo-radiation therapy
15. Known pregnancy or lactating
16. Inability to complete the study or required follow-up.

5. PRE-TREATMENT EVALUATION

Refer to Appendix III: Schedule of Study Assessments and Evaluations

The Baseline assessment is to include documentation of cancer staging as per FIGO staging criteria (see ***Appendix I***). The presence of relevant comorbidities and ECOG performance status is to be recorded. Physical exam will include the assessment of height and weight.

Other baseline assessments will include: a chest X-ray (or chest CT scan) and blood tests (creatinine and random blood sugar) within a 28 day period prior to randomization. Pelvic MRI as performed according to local centre practice will be documented and utilized for stratification at the time of randomization. Patients may or may not have had a contrast-enhanced CT scan of the abdomen and pelvis at the time of randomization. If a patient has had a contrast-enhanced CT scan of the abdomen and pelvis it must have been done within 6 weeks of randomization. Otherwise, the CT scan needs to be repeated (see ***Section 7.2*** for minimum standards and ***Appendix VI, item 6.*** for contrast-enhanced CT scan procedures).

The QoL questionnaires (EORTC QLQ-30 version 3 and EORTC QLQ-CX24, see ***Appendix IV***) and the questionnaire to measure health utilities (EQ-5D, see ***Appendix V***) are to be completed by the patient after informed consent has been obtained. It is preferable for the patient to complete these questionnaires prior to randomization, although this is not mandatory.

6. RANDOMIZATION

Patients will be allocated to one of two imaging groups:

Study Group	Intervention
1	CT Abdomen and Pelvis* + whole body PET-CT† (Experimental arm)
2	CT Abdomen and Pelvis* (Standard arm)

* Usual strategy is contrast-enhanced CT of abdomen and pelvis

† If contrast-enhanced CT of abdomen and pelvis has been done prior to randomization, then a low resolution CT is done with the PET. If a contrast-enhanced CT of abdomen and pelvis was performed within 6 weeks prior to randomization, it is not repeated.

6.1. Stratification

Eligible, consenting patients will be stratified by:

(1) Pre-randomization CT scan abdomen and pelvis *versus* no pre-randomization CT scan abdomen and pelvis

(2) Pre-treatment pelvic MRI *versus* no pelvic MRI

(3) Stage of cervical cancer (Stage IB through IIA *versus* Stage IIB through IVA)

(4) Treatment centre.

6.2. Randomization Procedure

Randomization will be conducted centrally by the OCOG Coordinating & Methods Centre (CMC) located at the Henderson Research Centre, Hamilton, Ontario. A minimization technique will be used to allocate patients in a 2:1 ratio (in favour of PET-CT) to receive either pre-treatment whole body PET-CT or no PET-CT scan. This will give the patient a greater chance to receive the experimental intervention with minimal loss of power. After confirmation of patient eligibility and documentation of written informed consent, stratification will be determined by the clinical centre and randomization will be completed by accessing the CMC's web-based Interactive Registration/Randomization System (IRIS). All patients must be registered before any study intervention and/or treatment is started.

7. STUDY INTERVENTION

The intervention should be performed within 4-6 weeks from the time of randomization. Women of child bearing potential must undergo a pregnancy test (serum or urine) prior to the study intervention. If the patient is pregnant, they must not undergo the PET-CT.

7.1. CT Abdomen and Pelivs + Whole-body PET-CT Group (Experimental arm)

Patients randomized to the experimental arm will undergo pre-treatment whole body ^{18}FDG PET-CT. The imaging strategy will start with a low-dose CT scan to enable definition of the axial field of view and for use in attenuation correction of the emission scan. This will be followed by acquisition of the FDG emission scan. In those patients who have not had a

conventional contrast-enhanced CT scan it would be feasible to subsequently conduct a contrast-enhanced diagnostic CT of the abdomen and pelvis[41] acquired with identical minimal standards to those patients in the CT abdomen and pelvis group. Some of the PET-CT scanners in the trial are capable of performing such CT imaging, but not all can. Hence the contrast-enhanced CT of the abdomen and pelvis can be performed on a separate CT machine (see Section 7.2). The details for the PET-CT + CT imaging protocol have been developed in conjunction with experienced nuclear medicine physicians, physicists and radiologists (see ***Appendix VI***). All patients will be required to fast prior to the PET scan and undergo regular monitoring of blood glucose levels by a glucometer prior to receiving the injection of ^{18}FDG.

7.2. CT Abdomen and Pelvis Group (Standard arm)

Patients randomized to the standard arm will have a conventional CT of the abdomen and pelvis. The minimum standards recommended for CT scans are as follows:

- 5 mm collimation
- helical or spiral scanner technology
- IV contrast in portal venous phase if no contraindications (all injections performed with a power injector at a rate of at least 2cc/s, and contrast dose should be 150ml of [270-300mgI/ml] iodine contrast agent or 2ml/kg up to 200ml). Oral contrast is water soluble, iodinated oral contrast such as MD Gastroview® (Mallinckrodt Inc.) or equivalent, not barium. Typically, a total dose of 600 mL of MD-Gastroview will be ingested.

7.3. Post-Imaging Diagnostic Confirmation

The **primary interpretation of lymph node metastases** should be based on visual criteria:

Lymph nodes ≤ 1 cm in short-axis diameter (per CT) should be considered positive if there is activity above that of the surrounding background.

Lymph nodes > 1 cm in short-axis diameter (per CT) should be considered positive if there is activity greater than blood pool activity. For this purpose, mediastinal blood pool activity can be used a as a reference regardless of location of node.

There should also be **recording of SUV measurements** in positive nodes.
SUV should be measured with an ROI to encompass at most 2/3 of the diameter of the node to minimize partial volume effects.

As there may be variability in SUV measurements between different PET centres and between different studies, 2 measurements of SUV of the liver should also be recorded as normal reference. A 3 cm diameter region of interest in the right lobe of the liver should be used for these 2 measurements.

7.4. Quality Assurance

When the OCOG program for the evaluation of PET in oncology commenced in 2002, a quality assurance (QA) program was put in place to establish standards for PET scanners, radioisotopes and interpretation of scans. Initially the PET scans were double read by a primary reader at the clinical centre and an external reader. Any discrepancies were resolved by consensus. In recent years, this process was modified because of the extensive experience gained in over 1600 patients. Currently intermittent checks are performed involving double reads (see ***Appendix VII***).

In addition for any new anatomic site (i.e. cervix), for which a new study is commenced, the first 10 patients at each PET participating centre will have their scans interpreted by a primary reader at the centre and a second external reader. Any discrepancies are resolved by consensus. Currently, a provincial physics quality assurance committee meets quarterly to ensure compliance with current guidelines for imaging.

Quality assurance may be performed to assess a sample of the diagnostic CT scans.

8. STUDY TREATMENT

8.1. Standard Radiation Therapy for Locally Advanced Cervical Cancer

The recommended standard radiation is described within this section. Management guidelines based on nodal staging is shown in Table 5. The final treatment plan will be determined by the treating investigator.

8.1.1. Pelvic Radiation

Whole pelvic external radiation will include 45-50 Gy in 1.8 Gy per fraction for 25 fractions over a period of five weeks. Intracavitary brachytherapy 35-40 Gy will be given in one to two implants low dose rate (LDR) or pulse dose rate (PDR) or 24-30 Gy intracavitary brachytherapy in 3-5 fractions high dose rate (HDR). Parametrial boost 5-10 Gy/1.8-2 Gy/3-5 fractions over 3-5 days to involved parametria will be added if the total dose to the sidewall is less than 60 Gy. Overall treatment time is not to exceed eight weeks.

8.1.1.1. External Irradiation

Patients will be simulated and treated in the supine position using immobilization devices as appropriate. CT planning will be performed in all patients to define tumour, clinical and planning target volumes.

Patients will receive 45 to 50 Gy external beam RT delivered to the pelvis in 25 fractions of 1.8 to 2 Gy using 10-25 MV photons. Treatments are to be delivered daily, five fractions per week and should be completed within 5 weeks ± 3 days. Patients should be treated with their bladder full. If intracavitary RT cannot be performed, shrinking field technique or boost with conventional radiotherapy (CRT)/intensity modulated radiation therapy (IMRT) should be performed to bring gross tumour volume with adequate margins to a minimum of 65 Gy. An attempt should be made to exclude all small bowel from treatment field after 50.4 Gy.

Whole Pelvis Field

The external RT target volume should encompass, with adequate margins, the primary cervical tumour and its gross extension and any grossly involved pelvic lymph nodes as well as possible microscopic extension to pelvic lymph nodes and the uterus. The tumour volume including gross nodal disease should be delineated on the digitally reconstructed radiographs (DRRs) if CT treatment planning is used.

AP/PA Pelvic Fields

The superior border will be through the L5-S1 interspace unless the target volume would not be encompassed adequately in a cephalad direction. In this case, a 2cm margin should be added to the highest level of pathologic abnormality, but should not be cephalad to the L-4/L-5 interspace. The lateral border will be 2cm beyond the lateral margins of the bony pelvis. The inferior border will be inferior to the obturator foramen or the lowest extension of disease with at least a 3cm margin. The inferior extent of cervical cancer or vaginal extension should be marked so that the inferior border of disease can be documented. Uninvolved normal tissues may be blocked although the position of the uterus should be contoured to ensure adequate coverage. Multi-leaf collimator (MLC) or custom Cerrobend blocks are acceptable for field shaping.

Lateral Pelvic Fields

The anterior border should be at the anterior symphysis pubis, and the posterior border will be through the posterior sacrum to include the cervical disease with a margin of 3-4cm. Superior and inferior borders will be the same as for the anterior and posterior fields. At least 3cm should not be blocked anterior to the L-5 vertebral body. Also the anterior two-thirds of the L-5 vertebral body should not be blocked. Posterior blocking should be designed such that gross disease is encompassed with at least a 2.5cm margin. The outer table of the sacrum should be blocked to protect the sacral plexus on lateral fields.

Common Iliac and Para-Aortic Fields

Pelvic and para-aortic regions will be treated to a total dose of 40-45 Gy in 5 weeks. Four-field technique (AP-PA and lateral opposed fields) or AP pair will be used. Patients will be treated once a day, 5 days a week with a daily fraction size of 1.8-2 Gy.

Superior border will be a transverse line between T12 and L1 for PA fields, and between L1 and L2 if common iliac nodes are involved. The inferior border will be as per description of pelvic fields above. Lateral border will be 2cm lateral to widest true pelvic diameter unless the distal 1/3 of the vagina is involved; in which case the medial inguinal lymph nodes should also be treated. Custom blocking will be used to shield small bowel and femoral heads while maintaining a margin of at least 1cm from common iliac and PA nodes and should not shield the obturator foramina.

Lateral Common Iliac and Para-Aortic Fields

Anterior border will be anterior to the symphysis pubis and at least 1cm anterior to common iliac or PA nodes. Posterior border: will be through the posterior sacrum to include the cervical disease with a margin of 3-4cm; blocking should split the T12-L5 vertebral bodies

to shield posterior soft tissue and may split the sacrum to provide adequate margin for presacral nodes; posterior blocking should be designed so that gross disease is encompassed by at least a 3cm margin. Custom blocking will be used to shield small bowel.

Dose Specification

The dose to the pelvis shall be calculated at the intersection of the axes of the 4 field box or at midplane of an AP pair. The dose to point B shall also be calculated using an off axis calculation (or identified on isodose distribution). The dose to central structures shall be the sum of the dose at the intersection of the axes of the 4 field box and the dose to point A from the intracavitary treatment. The dose to sidewall structures shall be calculated bilaterally and shall be the sum of the dose at the intersection of the axes from the 4-field box plus the intracavitary dose to each point B plus the contribution from the parametrial fields.

8.1.1.2 Pelvic Boost

Following common iliac or para-aortic RT to 40-45 Gy a pelvic boost of a further 5-10 Gy (total 50 Gy) in 1.8-2 Gy fractions may be used for patients with bulky central disease. Fields used will be according to guidelines for pelvic field above.

8.1.2. Intracavitary Brachytherapy

Low Dose Rate or Pulse-Dose Rate (LDR/PDR) Brachytherapy

Following the completion of whole pelvic RT the patient will receive 35 to 40 Gy to Point A by intracavitary implant. The patient may receive this in one or two applications at the discretion of the radiation oncologist. The first insertion should be performed promptly upon completion of whole pelvic RT. If two implants are contemplated, the second implant should be completed within three weeks of the completion of whole pelvic RT.

High Dose Rate (HDR) Brachytherapy

Total HDR brachytherapy dose to Point A will be 24-30 Gy: in 6-8 Gy fractions delivered in 3-5 weekly HDR brachytherapy procedures to deliver an LDR equivalent dose of 40 Gy. HDR brachytherapy should start week 3-5. When HDR brachytherapy begins, at least one insertion will be performed per week with no external beam therapy given on the day of the insertion. If the majority of the external beam radiation has been given, then two insertions per week could be done separated by at least 72 hours in order to complete all treatment with 8 weeks.

Brachytherapy Instruments

It is recommended that tandem and ovoids or tandem and ring be used for HDR brachytherapy, and tandem and ovoids or tandem alone for LDR/PDR brachytherapy. Image-guided brachytherapy will be permitted.

Determination of Normal Tissue Tolerance

In order to stay below an LDR equivalent of 70 Gy to the rectum (120 Gy) for the 3-5 HDR insertions, including the 45-50 Gy contribution from the external beam radiation, the rectum should receive less than 4.1 Gy for each HDR fraction of 6 Gy (68% of the prescribed dose to Point A). The dose to the bladder should be less than 4.6 Gy per each HDR fraction of 6

Gy (77% of the prescribed dose to Point A). As in LDR brachytherapy, every attempt should be made to deliver tumouricidal doses, even if the late responding tissues receive a slightly higher dose. Brachytherapy volumes and doses will be compared between the two intervention groups.

8.2. Standard Chemotherapy for Locally Advanced Cervical Cancer

Cisplatin 40 mg/m^2 (max 70 mg) IV given weekly for a total of six weekly cycles.

9. CONCOMITANT MEDICATION

9.1. Prohibited Concomitant Medication and Therapy

Aminoglycoside antibiotics (see ***Appendix VIII***) given before, with or after cisplatin may potentiate renal toxicity and should be avoided whenever possible.

10. EVALUATION DURING AND AFTER TREATMENT

Refer to Appendix III: Schedule of Study Assessments and Evaluations

10.1. Treatment Period Assessment

The usual practice at Ontario centres is for patients to undergo weekly blood tests (CBC plus differential, creatinine, and electrolytes) during chemo-RT treatment. However, the blood tests and their frequency will be left to the discretion of the local investigators.

10.2. Follow-up Assessment

Patient follow-up care will be at the discretion of the investigator at the local site. It is suggested that follow-up care be in accordance with the Cancer Care Ontario guidelines[42]. For the purposes of collection of study related data, patients will be followed for approximately 5 years. Assessments will be calculated from the date of randomization.

Patients will be assessed every 6 months (±14 days) for the first 2 years (Year 1 and 2). At each follow-up visit, patients will be required to return to the clinic to assess current disease status. ECOG performance status will also be assessed and documented. The two QoL questionnaires (EORTC QLQ-30 version 3 and QLQ-CX24) will be completed by the patient at 3, 6, 12, 18 and 24 months after randomization. These questionnaires can be either mailed to the patient or completed by the patient at a clinic visit *(*see ***Appendix IV****)*.

To perform the health economic evaluation, a limited set of questions on health care resource utilization and patient utility will be incorporated into the data collection. The health utility questionnaire (EQ-5D) will be completed by the patient at 3, 6, 12, 18 and 24 months after randomization. This questionnaire can be either mailed to the patient or completed by the patient at a clinic visit (see ***Appendix V***).

Information on a patient's cancer status (free of disease or not) and overall survival will be abstracted from their cancer centre or hospital records, or by contacting the family physician at least once a year for Years 3, 4 ,and 5.

On completion of the 5 years of follow-up required by this study, further assessment of the patient will be according to the oncologist's or local institutional practice; patients will not be restaged at the end of the 5-year follow-up period.

11. ADVERSE EVENTS

This study will be conducted in accordance with Health Canada regulatory requirements and ICH Good Clinical Practice guidelines. Adverse events (AEs) and Serious Adverse Events (SAEs) data will be reported and collected.

11.1. Adverse Event Definitions

An **Adverse Event (AE)** is defined as any untoward medical occurrence in a patient who is administered a drug or biologic (medicinal product) or using a medical device; the event does not necessarily have a causal relationship with this treatment. An AE can therefore be any unfavourable and unintended sign (including an abnormal laboratory finding), symptom, or disease temporally associated with the use of a medicinal (investigational) product, whether or not related to the medicinal (investigational) product. Each AE is to be classified by the Investigator as serious or non-serious.

A **Serious Adverse Event (SAE)** is any untoward medical occurrence that at any dose:

- results in death,
- is life-threatening (i.e., immediate risk of death),
- requires inpatient hospitalization or prolongation of existing hospitalization, except pre-planned hospitalizations required as part of a standard procedure or cancer treatment,
- results in persistent or significant disability / incapacity,
- is a congenital anomaly / birth defect
  OR
- is an important medical event, which is not immediately life threatening or requiring hospitalization, but may otherwise jeopardize the patient or may require intervention to prevent other outcomes specified in the above definition of SAE

An AE is **unexpected** when the nature or severity of the AE is not consistent with the applicable product information (i.e., Investigator's Brochure) for an unapproved investigational product or package insert/summary of product characteristics for an approved product. An AE is considered to be associated with the use of the drug if the attribution is classified as "Possible", "Probable" or "Very Likely".

11.2. Attribution Definitions

Not Related	An AE which is not related to the use of the study drug.
Doubtful	An AE for which an alternative explanation is more likely (e.g. concomitant medication, concomitant disease), and/or the relation with time suggests that a causal relationship is unlikely.
Possible	An AE which might be due to the use of the study drug. An alternative explanation (e.g., concomitant medication, concomitant disease) is inconclusive. The relationship in time is reasonable; therefore the causal relationship cannot be excluded.
Probable	An AE which might be due to the use of the study drug. The relationship in time is suggestive (e.g., confirmed by a de-challenge). An alternative explanation is less likely (e.g.,concomitant medication, concomitant disease).
Very Likely	An AE, which is listed as a possible adverse reaction and cannot be reasonably explained by an alternative explanation (e.g., concomitant medications, concomitant diseases). The relationship in time is very suggestive (e.g. it is confirmed by a de-challenge and a re-challenge).

11.3. Adverse Event Reporting Criteria

The investigational drug for this trial is ^{18}FDG. Currently, all clinical trials involving ^{18}FDG PET-CT require regulatory approval through Health Canada, hence, AEs and SAEs will be considered related to study drug if they are deemed to be related specifically to the administration of ^{18}FDG. **Since all procedures other than that directly related to ^{18}FDG PET-CT are standard of care, only AEs attributable to ^{18}FDG will be collected.**

Progression of cancer and complications related to cancer treatment (e.g. surgery, chemotherapy and/or radiotherapy) are expected and bear no relationship to ^{18}FDG, therefore they will not be documented as AEs or SAEs for the purpose of this trial. Deaths due to recurrent cancer are also expected and will not be reported as SAEs for the purpose of this trial. Deaths will be monitored by the Data Safety Monitoring Board.

AEs are to be recorded within the electronic CRF. Whenever possible, symptoms should be grouped as a single syndrome or diagnosis. The Investigator should specify the date of onset, grade, action taken with respect to Investigational Product, corrective treatment/therapy given, outcome and his/her opinion as to whether there is a reasonable possibility that the AE was related to the Investigational Product.

The severity of all AEs will be graded according to the NCI CTCAE, version 4.02. For each event, the highest severity grade attained since the last assessment period will be reported. If a CTCAE score does not exist, the Investigator should assess the event as Grade 1 (mild), Grade 2 (moderate), Grade 3 (severe), Grade 4 (life-threatening or disabling) or Grade 5 (causing death) to describe the maximum intensity of the AE.

11.4. Adverse Event Reporting Period

The AE reporting period for this study begins at the time of administration of ^{18}FDG and continues until 30 days after the administration of ^{18}FDG or at death if it occurs earlier.
In addition, any known untoward event of Grade 3 or greater severity that occurs subsequent to the AE reporting period that the Investigator assesses as Possibly, Probably or Very Likely related to the administration of ^{18}FDG should be reported as an SAE.

All AEs and SAEs must be followed until resolution unless, in the investigator's opinion, the condition is unlikely to resolve because of the subject's underlying disease.

Patients withdrawn from the study due to an AE will be followed until the AE has resolved. In the case of an SAE, the patient will be followed until clinical recovery or until progression has been stabilized or judged to be chronic.

11.5. Serious Adverse Event Reporting to OCOG

Adverse events considered to be **SERIOUS** *and* **UNEXPECTED** *and* **RELATED** (i.e., attributed as Possibly, Probably, or Very Likely related) to the administration of ^{18}FDG, must be reported by the Investigator to OCOG within 24 hours from the time when the clinical centre personnel became aware of the event. All other SAEs should be reported to OCOG within 5 days from the time when the clinical centre personnel became aware of the event.
Follow-up reports must be submitted to OCOG when new information becomes available and not later than 5 days after the clinical centre personnel became aware of the event. If an ongoing SAE changes in its intensity (Grade) or relationship to the Investigational Product, a follow-up SAE report should be sent immediately.

In the rare event that the Investigator does not become aware of the occurrence of an SAE immediately, the Investigator is to report the event to OCOG within 24 hours of notification of the event.

The occurrence of SAEs and follow-up information related to SAEs will be documented by the clinical centre using the OCOG SAE Form. Relevant source documentation should be included. Forms and source documentation must be submitted by confirmed facsimile transmission (fax) and the original mailed to OCOG.

SAE FACSIMILE TRANSMISSION:

1-905-575-2639

SAE MAILING ADDRESS:

OCOG
Henderson Research Centre, 1st Floor
711 Concession Street,
Hamilton, ON Canada
L8V 1C3

11.6. Serious Adverse Event Reporting to Health Canada

OCOG, acting as Sponsor, will be responsible for notifying Health Canada, Biologics and Genetic Therapies Directorate (BGTD) in an expedited manner of adverse events which are considered **SERIOUS** *and* **UNEXPECTED** *and* **RELATED** to the administration of ^{18}FDG (or for which a causal relationship with the administration of ^{18}FDG cannot be ruled out). For each event reported to Health Canada, OCOG will inform the DSMB.

Follow-up of SAEs as documented and submitted by the clinical centre on the OCOG SAE form will be forwarded to Health Canada by OCOG, where applicable.

11.7. Reporting SAEs to Local Research Ethics Boards

Investigators will be notified by OCOG of all SAEs that were reported to Health Canada and will be instructed to notify their local REB/IRB of the same, as per local REB/IRB requirements. The submission of these events by the investigator to their local REB/IRB should be completed upon receipt and not later than 30 days from the date of the correspondence from OCOG.

The Investigator will be responsible for reporting SAEs occurring at their local site to their local REB/IRB, as per local REB/IRB requirements.

12. STUDY OUTCOMES

12.1. Primary Outcome

The primary outcome is treatment delivered. Any patient who does not complete treatment as scheduled due to adverse events, withdrawal, or other reasons, will have treatment delivered defined as the treatment planned. The actual treatment delivered will be classified into one of the following three categories:

(1) standard pelvic chemo-radiotherapy of curative intent (i.e. not palliative care nor extended field radiation therapy). This will include all brachytherapy volumes and doses.

(2) more extensive chemo-radiotherapy of curative intent, such as EFRT, either with or without biopsy. EFRT includes any treatment extending outside the standard pelvic RT field as defined in Table 5, including para-aortic or upper common iliac lymph nodes, or distal vagina.

(3) therapy with palliative or non-curative intent for example, symptom management, chemotherapy, radiotherapy or both.

It is conceivable that by allowing patients with pre- randomization CT scans to be eligible for the study bias could be introduced. Patients who had distant metastases e.g. liver documented on CT scan would not be referred for the trial; whereas patients with a normal liver on CT prior to randomization would be referred to the trial and that CT would be considered as the "usual intervention". For patients who did not have a pre-randomization

CT and who were allocated to PET-CT, the contrast-enhanced CT could detect any liver metastases, thus favoring the PET arm. We feel that such a bias is unlikely because liver metastases is a rare occurrence in cervix cancer.

12.2. Secondary Outcomes

Secondary Outcomes are:

1. ***Event-free Survival (EFS)***: EFS is calculated from the date of randomization to the date of objective disease recurrence, or objective disease progression or death due to any cause following study treatment. The EFS of patients alive and without disease recurrence or progression at the time of analysis will be censored on the last clinic follow-up date. Suspicion of recurrent or progressive disease will be verified with further investigations as per the centre's practice. Objective disease recurrence or progression will be defined as a positive result on a biopsy of clinical disease or by disease progression on radiologic imaging.
2. ***Overall Survival (OS)***: OS is calculated as the date from randomization to the date of death due to any cause. Patients alive at the time of analysis will be censored on the last date the patient is confirmed to be alive.
3. ***Standardized Uptake Value (SUV)***: The SUVs of the PET-CT cervical cancer scans will be used to assess their ability to predict EFS and OS.
4. ***Economic and Quality of Life Analyses*** (see ***Appendices IV, V*** and ***IX***)

13. STATISTICAL CONSIDERATIONS

13.1. Statistical Analysis

The primary outcome is treatment delivered. Exact tests will be used to investigate a difference in the proportion of patients receiving standard pelvic chemo-radiotherapy of curative intent, more extensive chemo-radiotherapy of curative intent or therapy with palliative or non-curative intent both unadjusted (e.g. exact χ^2 test) and after adjusting for the stratification factors (e.g. logistic regression). A secondary analysis will explore the specific treatments delivered (e.g. brachytherapy dose and volume, parametrial boost doses, external radiation dose and volume, fractions, overall treatment duration, radiotherapy technique – IMRT versus conventional radiotherapy, chemotherapy dose and duration) using descriptive statistics.

The longer term question concerns the subsequent EFS and OS of patients following EFRT. If, as anticipated, the PET-CT strategy is able to generate a cohort of patients who have para-aortic nodal disease that is curable by EFRT, then the 5-year EFS and OS of patients following surgery would be expected to be higher for patients in the CT abdomen and pelvis + whole body PET-CT group *versus* the CT abdomen and pelvis group. EFS and OS will be estimated and plotted for each arm separately using Kaplan-Meier methods; the treatment arms will be compared using Cox proportional hazards regression models.

The prognostic ability of PET SUV of cervical cancer will be investigated as a risk factor for EFS and OS using Cox proportional hazards regression models amongst those patients

undergoing PET-CT only. Economic and Quality of Life analyses are described in Appendices IV, V and IX.

Some missing data is expected for economic outcomes and will be assumed to be missing at random. Missing data will be left as missing for other outcomes (e.g. baseline information) and descriptive statistics will be used to report the number of missing results. Missing data is not expected to occur for the primary outcome except in cases where patients withdraw from the study or rapidly deteriorate. In this case, the planned treatment will be used in lieu of treatment delivered.

All tests will be two-sided and based on an intent-to-treat analysis. A p-value of 0.05 or less will be considered statistically significant. In addition, 95% confidence intervals will be constructed for outcomes of interest. All data analysis will be performed using the latest versions of SAS (Cary, NC) and using StatXact (Boston, MA).

13.2. Sample Size and Feasibility

It is postulated that approximately 25% of patients having the CT of the abdomen and pelvis alone will receive either palliative care (~10%) or EFRT (~15%). The use PET-CT in addition to CT of the abdomen and pelvis would be considered valuable if the proportion of patients treated with either palliative care or EFRT increased to 45% (20% and 25% respectively). These estimates are derived using the estimates available from the previous smaller studies performed on the older PET scanners. Further, a 20% absolute difference was felt to be a clinically important difference and is slightly more conservative than the 50% relative change in treatment rate observed in the OCOG trial of PET in early lung cancer [37]. Based on these considerations, a trial with 288 patients (192 PET-CT, 96 CT abdomen and pelvis) will have over 90% power to detect a difference of this magnitude using the Fisher's exact test (α=0.05, two-sided).

The 2:1 randomization for this trial allows us to gain additional understanding of the potential effect of ^{18}FDG PET-CT plus CT of the abdomen and pelvis to CT of the abdomen and pelvis alone, which is currently the standard of care in patients with locally advanced cancer of the cervix. Using this unbalanced allocation will increase the number of patients who receive ^{18}FDG PET-CT and could potentially help with recruitment. We feel this is ethical as preliminary data indicate the use of PET-CT may be better than CT abdomen and pelvis alone and it is unlikely that PET-CT will be detrimental to the individual patient. (Although unlikely to be detrimental, it is unclear that there is a benefit and even if a benefit exists, whether it is sufficient to warrant extensive use of PET-CT, hence the rationale for the study).

With a traditional 1:1 randomization, 128 patients per arm (256 in total) would be required. By using the 2:1 randomization scheme, 32 additional patients will need to be recruited to a total of 288 subjects. However, at the end of the study, 64 more patients will have undergone PET-CT (consequently, 32 fewer patients will have CT abdomen and pelvis alone) compared to 1:1 randomization, while maintaining α=0.05 and 90% power for a two-sided test. By gaining additional information on 64 more patients with PET-CT, we will have better estimates of the true change in management rate due to PET-CT, and of EFS and OS resulting from the use of PET-CT. Further, these additional patients will give us more

information to investigate the effect of PET SUV on EFS in this patient population, which is an important scientific question. Thus, it is felt the small increase in total sample size is warranted.

In Ontario for the fiscal period 2003-2004, there were 513 women who presented with cervical cancer: 220 did not receive surgery and 186 had a radiation oncology consult and treatment planning. These data have not changed significantly since then. Thus, a conservative estimate of recruitment is approximately 120 subjects per year[43,44]. Based on these considerations, and an anticipated 6 month startup period, recruitment will be complete in approximately 2.5 years from study commencement. The Investigators involved in this trial include gynecologic oncologists and radiation oncologists who have a strong track record of recruitment to clinical trials.

Steps will be put in place to minimize losses to follow-up. Clinical trials staff will pursue rebooking any appointments that are not kept. Losses to follow-up in trials are known to be lower than in standard care. In OCOG's PET trials, the loss to follow-up rate is very low. There are a certain small number of patients who do not undergo PET due to comorbid conditions or other outside circumstances.

13.3. Analysis Sets

All patients who are randomized will be included in the Full Analysis set. All patients who complete the baseline QoL and at least one follow-up QoL questionnaire will be included in the QoL Analysis set.

Analysis of the treatment delivered, EFS and OS will be based on the Full Analysis set with patients included in groups according to their randomized allocation (i.e. intention-to-treat principle). A secondary analysis of treatment delivered, EFS and OS will be performed on the protocol evaluable analysis set, defined as the subset of patients in the Full Analysis set excluding patients with disease which has spread to distant organs. Analysis of QoL and economic outcomes will be based on the QoL Analysis set. Investigation of the prognostic ability of PET SUVs will be based on the patients who underwent PET-CT.

13.4. Health Related Quality of Life Data

Overall QoL will be assessed using the EORTC QLQ-C30 version 3, and disease-specific QoL using the EORTC QLQ-CX24. The mean and standard deviation of each QoL domain will be calculated at each time point, along with changes from baseline. The profile of the QoL scores will be plotted and compared between the two arms. A repeated measures regression model with an unstructured covariance structure will be used to evaluate the effect of the intervention longitudinally. Other covariance structures may be used if necessary. Descriptive statistics, plots and tables will also be used to explore for an effect on QoL between CT of the abdomen and pelvis + PET-CT and CT of the abdomen and pelvis alone over time. Effect of treatment regimen on QoL will also be investigated.

13.5. Health Economic Data

A description of the economic analysis is described in ***Appendix IX***.

13.6. Planned Interim Analysis

One planned interim efficacy analysis will be performed after 50% of patients have been accrued. The Haybittle-Peto approach will be used, such that if the proportion of patients receiving standard pelvic chemo-radiation following CT abdomen and pelvis + PET-CT is significantly less (one-sided $\alpha=0.001$) than the proportion of patients receiving standard pelvic chemo-radiation following CT abdomen and pelvis alone, then the Data Safety Monitoring Board (DSMB) will be expected to recommend to the Steering Committee that the trial should be discontinued early. In addition to the interim analysis based on efficacy, the DSMB will review accumulating safety data annually, starting approximately twelve months after study commencement. The Steering Committee will make the final decision about study continuation based on these recommendations.

14. CENTRAL ADJUDICATION

A Central Adjudication Committee, composed of independent experts in oncology, will be available to review any disagreements about whether treatment was standard chemo-radiotherapy of curative intent, more extensive therapy of curative intent or non-curative therapy.

15. STUDY SIGNIFICANCE

Cervical cancer is the second leading cause of cancer for women in their reproductive years. Currently, women with locally advanced cervical cancer receive chemo-RT. PET-CT has the potential to more accurately stage the cancer than CT or MRI by detecting tumour in para-aortic nodes and/or extra pelvic sites. In the former situation, RT will be altered by extending the fields to include the para-aortic nodes and the disease is potentially curable. In the latter situation the patient is counseled concerning the palliative nature of her disease and a long treatment course that is not curative is avoided. This study will address whether PET-CT adds a clinically meaningful difference in care. The economic analysis will evaluate the incremental cost associated with adding PET-CT to the current strategy of staging investigations. In addition, the results will inform policy makers on the value of adding PET-CT in this clinical situation.

16. ETHICAL AND REGULATORY STANDARDS

This clinical trial will be conducted in accordance with the recommendations guiding physicians in biomedical research involving human patients adopted by the 18th World Medical Assembly, Helsinki, Finland 1964 and later revisions or the laws and regulations of the country, whichever provide the greater protection for the individual.

This clinical trial will be conducted in compliance with the ICH guidelines for Good Clinical Practice and will adhere to national laws and regulations of the country in which the study is performed.

Personnel involved in conducting this clinical trial will be qualified by education, training and experience to perform their respective tasks.

16.1. Informed Consent

OCOG, as Sponsor, will provide each clinical centre with a sample Informed Consent Form. The Informed Consent Form used by the local Investigator for obtaining the patient's informed consent must be reviewed and approved by OCOG prior to submission to the appropriate Ethics Committee [Research Ethics Board (REB) or Institutional Review Board (IRB)] for approval/favourable opinion.

It is the responsibility of the local Investigator or a person designated by the local Investigator and under the Investigator's responsibility, to provide each potential study patient, prior to inclusion in the study, full and adequate verbal and written information regarding the objectives and procedures of the study and the possible risks involved. The patient must be informed about their right to withdraw from the study at any time. The patient must be allowed adequate time to make an informed decision.

Prior to a patient's participation in the study, the locally approved written Informed Consent Form must be signed, name filled in and personally dated by the patient or by the patient's legally acceptable representative, and by the person who conducted the informed consent discussion. A copy of the signed and dated written consent form document and any other written information should be provided to the patient.

16.2. Research Ethics Board (REB) or Institutional Review Board (IRB)

Prior to the commencement of the study, the Investigator must submit this clinical trial protocol, the Informed Consent Form document, Investigator Brochure or product labeling information, recruitment materials/process, patient questionnaires, and any other written information to be provided to study patients to the appropriate Ethics Committee (REB/IRB) and is required to forward to OCOG a copy of the written and dated approval/favourable opinion signed by the Chairman with Ethics Committee (REB/IRB) composition.

The clinical trial (study number, clinical trial protocol title, version number and version date), the documents reviewed (clinical trial protocol, Informed Consent Form, Investigator's Brochure, etc.) and the date of review should be clearly stated on the written (REB/IRB) approval/favourable opinion.

During the clinical trial, any amendments or modification to the study protocol and/or Informed Consent Form document, as issued by OCOG, must be submitted to and approved by the local Ethics Committee (REB/IRB). The Ethics Committee (REB/IRB) should also be informed of any event likely to affect the safety of patients or the continued conduct of the study. Updates to the Investigator's Brochure should be sent to the Ethics Committee (REB/IRB).

Annual re-approval is required for as long as the study is open to patient accrual and/or patients are being followed and evaluated.

The Ethics Committee (REB/IRB) must be informed when the study is closed or has been suspended.

17. RESPONSIBILITIES OF THE INVESTIGATOR

One Qualified Investigator (QI) will oversee the trial at each clinical centre. The QI undertakes to perform the study in accordance with this clinical trial protocol, ICH guidelines for Good Clinical Practice and the applicable national regulations and local REB/IRB requirements.

The QI may appoint other individuals as he/she deems appropriate to assist in the conduct of the study. All appointed designates will be listed and provided to OCOG (Sponsor). The appointed designates will be supervised by and under the responsibility of the QI.

For the purpose of ensuring compliance with the clinical trial protocol, ICH GCP and applicable regulatory requirements, the QI agrees to permit auditing by or on the behalf of OCOG and inspection by applicable regulatory authorities. The Investigator agrees to allow the auditors/inspectors to have direct access to his/her study records for review.

The QI must maintain confidential study documentation and ensure the retention of these study documents as per national regulations. The Investigator must notify OCOG (Sponsor) prior to destroying any essential documents following the completion or discontinuation of the clinical trial. If the QI can no longer ensure retention of the study documentation, the QI is required to inform OCOG (Sponsor) to arrange the transfer of the relevant records to a mutually agreed upon designee.

17.1. Source Document Requirements

The OCOG Coordinating and Methods Centre will complete Case Report Form (CRF) verification by obtaining and reviewing local source documents, except for the pre-identified source data directly recorded in the CRF. The Informed Consent Form will include a statement by which the patient allows the Sponsor's authorized personnel, the Ethics Committee (REB/IRB), and the regulatory authorities to have direct access to source data which support the data on the CRFs. Such personnel, must maintain confidentiality according to privacy legislation.

17.2. Case Report Forms (CRFs)

It is the responsibility of the QI to maintain adequate and accurate CRFs designed to record the disease status, treatment, all observations, and other pertinent data to the research on each enrolled study participant. Data reported on the CRF that are derived from source documents must be consistent with the source documents.

Clinical centres will use Electronic Data Capture (EDC) to submit study data to OCOG. Electronic CRFs will be prepared for the data collection requirements except for fields specific to SAEs and patient questionnaires, which will be reported on paper forms. Paper CRFs must be completed in ink. Subjects are to be identified by subject study number, initials and date of birth.

When paper CRFs are utilized, data recorded must be neat and legible to ensure accurate interpretation of data. A correction, prior to submission of the CRF to OCOG, must be made

by striking through the incorrect entry with a single line and entering the correct information beside the incorrect entry. The correction must be dated and initialed by the person making the correction and must not obscure the original entry.

The completed CRF must be promptly reviewed, signed and dated. For EDC, review and approval/signature is completed electronically through an EDC tool.

Once the submitted paper CRFs and/or electronic study data are received at OCOG, data verification may result in additional requests to clarify or correct the data. For paper CRFs, these requests with their responses will be appended to the paper CRFs held by the Investigator and by OCOG. EDC queries will be tracked and archived electronically.

18. CONFIDENTIALITY

All information disclosed or provided by OCOG, or produced during the clinical trial, including, but not limited to, the clinical trial protocol, the Investigator's Brochure, the CRFs, operations manuals and the results obtained during the course of the clinical trial, is confidential. The QI and any person under his/her authority agrees to undertake to keep confidential and not to disclose the information to any third party without the prior written approval of OCOG. This excludes the required REB/IRB submission.

19. CLINICAL TRIAL PROTOCOL AMENDMENTS

Investigators should not implement any deviation from, or changes to the clinical trial protocol without written authorization from OCOG (Sponsor), prior review and documented written approval from their local Ethics Committee (REB/IRB), unless the safety of the study patient is in jeopardy.

An amendment may require a change to the Informed Consent Form. The Investigator must receive a REB/IRB approval/favourable opinion of the revised Informed Consent Form prior to the implementation of the change.

20. STUDY ORGANIZATION

20.1. Steering Committee

The Steering Committee will be responsible for the overall conduct of the trial, including the design, execution, analyses, reporting and will also be responsible for the assignment of responsibilities to other study committees. The Steering Committee will hold the primary responsibility for publication of the study results. This Committee will convene on a regular basis by teleconference or meetings at least every six months to address policy issues and to monitor study progress, execution and management and to review the reports from the DSMB. A list of the Steering Committee members is maintained by OCOG.

Drs. Laurie Elit and Anthony Fyles as Co-Principal Investigators along with OCOG will be responsible for supervising all aspects of the study including working with individual Investigators at the various clinical centres. Dr. Douglas Coyle will oversee the economic component of the study.

20.2. Data Safety Monitoring Board

The independent Data Safety Monitoring Board (DSMB) will review accumulating safety data, provided to them through the OCOG CMC, at least annually starting approximately 12 months after study commencement. In addition, the DSMB will review efficacy data at the one scheduled interim efficacy analysis which will occur after 50% of patients have been accrued. Members of the DSMB will be experts in the fields of clinical trial methodology and oncology. Refer to Section 13.7 for early stopping guidelines.

20.3. Study Coordination

The OCOG Coordinating and Methods Centre (CMC), located at the Henderson Research Centre in Hamilton, Ontario, Canada will be responsible for the overall study management including implementation and coordination of the protocol logistics, patient allocation, data management and statistical analysis. Web based randomization will be performed by the clinical centres utilizing the CMC's Interactive Registration/Randomization System (IRIS). A secure and confidential electronic study database will be maintained by the CMC. Data collection will be performed via an EDC system. The CMC's Online Remote Collection of Clinical Information and Data (ORCCID) system incorporates a clinical database, data query process, visit completion tracking and SAE tracking to ensure data is complete, accurate, of high quality and is submitted according to required timelines. Designated clinical centre personnel will be provided with member access to OCOG's website to obtain study specific documents, study manuals, monthly reports and newsletters.

An in house Ethics & Regulatory Affairs Officer will complete and submit the required applications to Health Canada and facilitate the local REB/IRB applications, ensuring required start-up documentation is obtained from each clinical centre prior to centre activation.

In addition, the CMC at OCOG will provide methodological and administrative support to all study committees, investigators and other study personnel.

20.4. Central Adjudication Committee (CAC)

A Central Adjudication Committee comprised of independent experts in the area of gynecologic oncology and radiation oncology will be responsible for assessing whether patient management has been altered as a result of up or down staging based on the result of PET imaging. The CMC at OCOG will provide support to the CAC, establishing a process for the collection, review and documentation of the adjudication items. Those performing adjudication will not be involved in the treatment of study patients.

21. SCIENTIFIC REPORTING AND PUBLICATION

The clinical trial protocol was developed by the Principal Investigator(s) and study Steering Committee, with the assistance of OCOG.

The Steering Committee is responsible for the scientific reporting, publishing and/or presentation of the study results. Authorship will be determined by the Steering Committee and will be guided by the extent of participation in the development of the protocol, accrual of patients to the protocol, involvement in the study analysis and the drafting of the final manuscript. Results of the study will be disseminated through publications and presentations at international meetings. Any other publication or presentation related to the study and the results by any investigator or participant must receive prior approval from the Steering Committee. No other publication or presentation is permitted before the primary publication or presentation by the Steering Committee.

The information developed during the conduct of this clinical study is considered confidential.

22. REFERENCES

1 Schiffman MH, Latest HPV findings: some clinical implications. Contemporary Ob/Gyn 38,27-40;1993

2 Marrett L, Dryer D, Ellison L, Logan H, Mery L, Morrison H, Schachter B. Canadian Cancer Statistics 2008 Canadian Cancer Society/National Cancer Institute of Canada. Pg 18,20

3 Health Canada. Cervical cancer screening in Canada: 1998 surveillance report.

4 Elit L, Krzyzanowska M, Saskin R, Razzaq, A., Kwon J, Bierman A. Equity Indicators for cervical cancer prevention in Ontario women. Results from the POWER (**P**roject for an **O**ntario **W**omen's Health **E**vidence-Based **R**eport Card) Study JOGC 30:5(S2);S30

5 Benedet JL, Odicino F, Maisonneuve P, Beller U, Creasman WT, Heintz APM, Ngan HYS, Pecorelli S. Carcinoma of the cervix Uteri in Int J Gyn Ob 83 (S1)Oct 2003;41-78

6 Pecorelli S, Zigliani L, Odicino, F. Revised FIGO staging for carcinoma of the cervix in Int J Gyn Ob 105 (S2) May 2009;107-108

7 Lukka, H., Hirte, H., Fyles, A., Thomas, G., Elit, L., Johnston, M., Fung Kee Fung, M., Browman, G. Concurrent Cisplatinum-based Chemotherapy plus Radiotherapy for cervical cancer – a meta-analysis. Clin Oncol 2002;14(3):203-212

8 Barbera, L., Paszat, L., Thomas, G., Covens, A., Fyles, A., Elit, L., Qui,F. The Rapid Uptake of Chemotherapy for Cervix Cancer Patients treated with curative radiation. *Int J Radiat Oncol Biol Phys* 2006;Apr1:64(5):1389-94

9 Randall M, Michael H, Vermorken J, Stehman F. Chapter 22 Uterine Cervix in Principles and Practice of Gynecologic Oncology 4th ed. Ed Goskins WJ, Perez CA, Young RC, Barakat R, Markman M, Randall M. Lippincott Williams and Wilkins C2005 743-822

10 Heller PB, Malfetano JH, Bundy BN. Clinical pathologic study of stage 2B, 3, 4A carcinoma of the cervix: extended diagnostic evaluation for paraaortic node metastasis (a GOG study). Gynceol Oncol 1990;38;425

11 Hacker NF. Cervical Cancer Chapter 9. in Practical Gynecologic Oncology Ed Berek JS and Hacker NF. 4th ed. Lippincott Williams and Wilkins, Philadelphia, C2005 Pg 347

12 Warburg OWFNE. On the metabolism of tumors in the body. Metabolism of tumors. London: Constable, 1930: 254-270.

13 Som P, Atkins HL, Bandoypadhyay D, Fowler JS, MacGregor RR, Matsui K et al. A fluorinated glucose analog, 2-fluoro-2-deoxy-D-glucose (F-18): nontoxic tracer for rapid tumor detection. J Nucl Med 1980; 21(7):670-675.

14 Hatanaka M. Transport of sugars in tumor cell membranes. Biochim Biophys Acta 1974; 355(1):77-104.

15 Nolop KB, Rhodes CG, Brudin LH, Beaney RP, Krausz T, Jones T et al. Glucose utilization in vivo by human pulmonary neoplasms. Cancer 1987; 60(11):2682-2689.

16 Rigo P, Paulus P, Kaschten BJ, Hustinx R, Bury T, Jerusalem G et al. Oncological applications of positron emission tomography with fluorine-18 fluorodeoxyglucose. Eur J Nucl Med 1996; 23(12):1641-1674.

17 Cohade C, Osman M, Leal J, Wahl RL. Direct comparison of (18)F-FDG PET and PET/CT in patients with colorectal carcinoma. J Nucl Med 2003; 44(11):1797-1803.

18 Delbeke D, Martin WH. PET and PET-CT for evaluation of colorectal carcinoma. Semin Nucl Med 2004; 34(3):209-223.

19 Antoch G, Saoudi N, Kuehl H, Dahmen G, Mueller SP, Beyer T et al. Accuracy of whole-body dual-modality fluorine-18-2-fluoro-2-deoxy-D-glucose positron emission tomography and computed tomography (FDG-PET/CT) for tumor staging in solid tumors: comparison with CT and PET. J Clin Oncol 2004; 22(21):4357-4368

20 Clarke JC. PET/CT "Cometh the hour, cometh the machine?". Clin Radiol 2004; 59(9):775-776.

21 Kapoor V, McCook BM, Torok FS. An introduction to PET-CT imaging. Radiographics 2004; 24(2):523-543.

22 Wahl RL. Why nearly all PET of abdominal and pelvic cancers will be performed as PET/CT. J Nucl Med 2004; 45 Suppl 1:82S-95S.

23 Havrilesky L, Kulasingam SL, Matchar DB, Myers ER. FDG-PET for management of cervical and ovarian cancer. Gyn Onc 2005;97:183-191

24 Selman TJ, Mann C, Zamora J, Appleyard TL, Khan K. Diagnostic accuracy of tests for lymph

node status in primary cervical cancer: a systematic review and meta-analysis. CMAJ. 2008 Mar 25;178(7):855-62
25 Wright JD, Dehdashti F, Herzog TJ et al. Preoperative lymph node staging of early stage cervical carcinoma by [18F}-Fluor-2-Deoxy-D-Glucose-Positron emission tomography. Cancer 2005;104-11:2484-2491
26 Yildirim Y, Sehirali S, Avci ME, Yilmaz C et al Integrated PET/CT for the evaluation of para-aortic nodal metastasis in locally advanced cervical cancer patients with negative conventional CT findings. Gynecol Oncol. 108 (2008) 154-159
27 Tsai CS et al. Preliminary report of using FDG-PET to detect extrapelvic lesions in cervical cancer patients with enlarged pelvic lymph nodes on MRI/CT. Int J Radiat Oncol Biol Phys. 2004 Apr 1;58(5):1506-12
28 Roh JW, Seo SS, Lee S et al. Role of positron emission tomography in pretreatment lymph node staging of uterine cervical cancer: a prospective surgicopathologic correlation study. Eur J Cancer 2005;41(14):2086-92
29 Grigsby PW, Siegel BA, Dehdashti F. Lymph node staging by positron emission tomography in patients with carcinoma of the cervix. *J Clin Oncol* 2001 Sep 1;19(17):3745-9
30 Yen TC et al. Value of dual-phase 2-fluoro-2-deoxy-d-glucose positron emission tomography in cervical cancer. J Clin Oncol. 2003 Oct 1;21(19):3651-8
31 Bjurberg M, Kjellen E, Ohlsson T et al. FDG-PET in cervical cancer: staging, restaging and followup. Acta Obstet Gynecol Scand 2007;86(11):1385-91
32 Kidd EA, Siegel BA, Dehdashti T, Grigsby PW. The standardized uptake value for F-18 fluorodeoxyglucose is a sensitive predictive biomarker for cervical cancer treatment response and survival. Cancer, 2007 Oct 15;110(8):1738-44
33 Xuw F, Lin L, Dehdashti F et al. F-18 fluorodeoxyglucose uptake in primary cervical cancer as an indicator of prognosis after radiation therapy. Gynecol Oncol 101 (2006) 147-151
34 Yen T, See L, Lai C et al. 18F-FDG uptake in squamous cell carcinoma of the cervix is correlated with glucose transporter 1 expression. J Nuclear Med 45(1):2004
35 Yoshida Y, Kurokawa T, Kawahara K et al. Metabolic monitoring of advanced uterine cervical cancer neoadjuvant chemotherapy by using [F-18}-Fluorodeoxyglucose positron emission tomography: preliminary results in three patients. Gyn Oncol 95 (2004) 597-602
36 Haie C, Pejovic MH, Gerbaulet A. Is prophylactic para-aortic irradiation worthwhile in the treatment of advanced cervical carcinoma? Results of a controlled clinical trial of the EORTC radiotherapy group. Radiother Oncol 1988;11;101-112
37 Maziuk D, Darling GE, Inculet RI, Gulenchyn K, Driedger AA, Ung YC, Miller JD, Koru-Sengul T, Evans WK, Levine MN. A randomized controlled trial (RCT) of 18F-fluorodeoxyglucose (FDG) positron emission tomography (PET) versus conventional imaging (CI) in staging potentially resectable non-small cell lung cancer (NSCLC). Clin Oncol 26: 2008 (May 20 suppl; abstr 7502).
38 Pritchard KP, Julian J, McCready D, Holloway C, Gulenchyn K, Hodgson N, Lovrics P, Down N, George R, Levine MN. A prospective study evaluating 18F-Fluorodeoxyglucose (18FDG) positron emission tomography (PET) in the assessment of axillary nodal spread in women undergoing sentinel lymph node biopsy (SLNB) for breast cancer. J Clin Oncol 26: 2008 (May 20 suppl; abstr 533).
39 Gulenchyn K, Maziak D, Darling G et al….Ontario oncology trials to evaluate the clinical and economic impact of 18-FDG PET. J Nucl Med 2008;49(Supp 1)41P
40 Kizer NT, Zighelboim I, Case AS, Dewdney SB, Thaker PH, Massad LS. The role of PET/CT in the management of patients with cervical cancer: practice patterns of the members of the Society of Gynecologic Oncologists. Gyn Onc 2009;114(2):310-4.
41 Wong TZ, Paulson EK, Nelson RC, Patz EF, Coleman RE. Practical approach to diagnostic CT Combined with PET. AJR 188;2007, 622-629
42 Elit L, Fyles A, Devries-Aboud M, Oliver T, Fung-Kee-Fung M, and the Gynecology Cancer Disease Site Group. Follow-up for women after treatment for cervical cancer: A systematic review. Accepted Gyn Onc 2009
43 Elit L, Schultz S, Prysbysz R, Barbera L., Saskin R, Gunraj N, Watson D, Urbach D. Patterns of Care for the Initial Management of Cervical Cancer in Ontario. In: Ontario Cancer Surgical Atlas ed M. Siminovic M, Urbach D. ICES and CCO, 2008
44 Benedet JL, Odicino F, Maisonneuve P, Beller U, Creasman WT, Heintz APM, Ngan HYS,

Pecorelli S. Carcinoma of the cervix Uteri: annual report on the results of treatment in gynecological cancer. J Epidemiol Biostat 2001;6:5-44
45 Ontario Case Costing Initiative, 2009. Available from http://www.occp.com/ [accessed January 2009]
46 Ministry of Health and Long-term Care of Ontario. Schedule of Benefits for Physician Services under the Health Insurance Act effective June 3, 2008
Available from: URL:
http://www.health.gov.on.ca/english/providers/program/ohip/sob/physserv/physserv_mn.html
[accessed January 2009]
47 Baladi JF. A guidance document for the cost process. Version 1.0. CCOHTA 1996
48 Brooks R. EuroQol: the current state of play. Health Policy. 1996;37(1):53-72
49 Brazier J, Deverill M. Obtaining the Q in QALYs: A comparison of five multi attribute scales. SHEG Discussion Paper 99/1.1999
50 Dolan P. Modelling valuations for EuroQol health states. Med Care. 1997;35:1095-108
51 Canadian Agency for Drugs and Technologies in Health. Guidelines for the economic evaluation of health technologies: Canada. 3rd ed. Ottawa: The Agency; 2006
52 Chaudhary MA, Stearns SC. Estimating confidence intervals for cost effectiveness ratios: an example from a randomized trial. Stat Med 1996;15:1447-58
53 Campbell MK, Torgerson DJ, Bootstrapping: estimating confidence intervals for cost-effectiveness ratios, QJM 1999:92; 177-182
54 van Hout BA, Al MJ, Gordon GS, et al. Costs, effects and C/E ratios alongside a clinical trial. Health Econ 1994; 3 (5): 309-19

APPENDIX I: CARCINOMA OF THE CERVIX: FIGO NOMENCLATURE (2009)

Stage I	The carcinoma is strictly confined to the cervix (extension to the corpus would be disregarded).
Stage IA	Invasive carcinoma that can be diagnosed only by microscopy. All macroscopically visible lesions – even with superficial invasion – are allotted to stage 1B carcinomas. Invasion is limited to a measured stromal invasion with a maximal depth of 5.0 mm and a horizontal extension of ≤7.0mm. Depth of invasion should not exceed 5.0mm from the base of the epithelium of the original tissue-superficial or glandular. The involvement of vascular space – venous or lymphatic – should not change the stage allotment.
Stage IA1	Measured stromal invasion of ≤3.0mm in depth and extension of ≤7.0mm
Stage IA2	Measured stromal invasion of >3.0mm and not >5.0 mm with extension of not >7.0mm
Stage IB	Clinically visible lesions limited to the cervix or preclinical cancers greater than stage 1A.
Stage IB1	Clinically visible lesions ≤ 4.0cm in size
Stage IB2	Clinically visible lesions > 4.0cm in size
Stage II	Carcinoma extends beyond the cervix, but has not extended onto the pelvic wall; the carcinoma involves the vagina, but not as far as the lower third
Stage IIA1	Clinically visible lesion ≤ 4.0 cm in greatest dimension with involvement of less than the upper two-thirds of the vagina.
Stage IIA2	Clinically visible lesion >4.0 cm in greatest dimension with involvement of less than the upper two-thirds of the vagina.
Stage IIB	Obvious parametrial involvement
Stage III	The carcinoma has extended onto the pelvic wall; on rectal examination, there is no cancer-free space between the tumor and the pelvic wall; the tumour involves the lower third of the vagina; all cases with a hydronephrosis or a nonfunctioning kidney are included, unless they are known to be due to another cause.
Stage IIIA	No extension onto the pelvic wall, but involvement of the lower one-third of the vagina
Stage IIIB	Extension onto the pelvic wall and/or hydronephrosis or nonfunctioning kidney
Stage IV	The carcinoma has extended beyond the true pelvis or involves (biopsy proven) the mucosa of the bladder or rectum. A bullous edema, as such, does not permit a case to be allotted to Stage IV.
Stage IVA	Spread of the growth to adjacent organs. Invades mucosa of bladder or rectum and/or extends beyond true pelvis
Stage IVB	Spread to distant organs

Clinical Diagnostic Staging

Staging of cervical cancer is based on clinical evaluation; therefore, careful clinical examination should be performed in all cases, preferably by an experienced examiner and under anaesthesia. The clinical staging must not be changed because of subsequent findings. When there is doubt as to which stage a particular cancer should be allocated, the earlier stage is mandatory. The following examinations are permitted: palpation, inspection, colposcopy, endocervical curettage, hysteroscopy, cystoscopy, proctoscopy, intravenous urography, and X-Ray examination of the lungs and skeleton. Suspected bladder or rectal involvement should be confirmed by biopsy and histologic evidence. Conization or amputation of the cervix is regarded as a clinical examination. Invasive cancers so identified are to be included in the reports. Findings of optional examinations (e.g, lymphangiography, arteriography, venography, laparoscopy, ultrasound, CT scan and MRI) are of value for planning therapy but, because these are not generally available and the interpretation of results is variable, the findings of such studies should not be the basis for changing the clinical staging. Fine-needle aspiration of scan detected suspicious lymph nodes may be helpful in treatment planning.

Postsurgical Treatment-Pathologic Staging

In cases treated by surgical procedures, the pathologist's findings in the removed tissues can be the basis for extremely accurate statements on the extent of disease. The findings should not be allowed to change the clinical staging, but should be recorded in the manner described for the pathologic staging of disease. The TNM nomenclature is appropriate for this purpose. Infrequently it happens that hysterectomy is carried out in the presence of unsuspected extensive invasive cervical carcinoma. Such cases cannot be clinically staged or included in therapeutic statistics, but it is desirable that they be reported separately. As in all gynecological cancers, staging is determined at the time of the primary diagnosis and cannot be altered, even at recurrence.

Only if the rules for clinical staging are strictly observed will it be possible to compare results among clinics and by differing modes of therapy.

APPENDIX II: ECOG PERFORMANCE STATUS*

Grade	Description
0	Fully active, able to carry on all pre-disease performance without restriction
1	Restricted in physically strenuous activity but ambulatory and able to carry out work of a light or sedentary nature, e.g., light house work, office work
2	Ambulatory and capable of all selfcare but unable to carry out any work activities. Up and about more than 50% of waking hours
3	Capable of only limited selfcare, confined to bed or chair more than 50% of waking hours
4	Completely disabled. Cannot carry on any selfcare. Totally confined to bed or chair
5	Dead

* As published in Oken MM, Creech RH, Tormey DC, Horton J, Davis TE, McFadden ET, Carbone PP: Toxicity And Response Criteria Of The Eastern Cooperative Oncology Group. *Am J Clin Oncol* 5:649-655, 1982.

APPENDIX III: SCHEDULE OF STUDY ASSESSMENTS AND EVALUATIONS

Assessments & Tests	Screening Baseline	STUDY INTERVENTION	Treatment	Follow-Up				
				Year 1	Year 2	Year 3	Year 4	Year 5
**Informed Consent**	X							
**Demographics**	X							
**FIGO Staging**	X							
Comorbidities	X							
**Physical Exam:**<br>• Height<br>• Weight	 X<br>X							
**ECOG Perf. Status**	X			Every 6m	Every 6m			
**Disease Status**				Every 6m	Every 6m			
**EFS and OS**						Annually	Annually	Annually
**Radiology:**<br>• CT abdomen/pelvis<br>• CXR or CT chest<br>• MRI pelvis	 X[*]<br>X[1]<br>X[#]							
**Lab Testing:**<br>• CBC + differential<br>• Creatinine<br>• Random blood sugar<br>• Electrolytes:<br>- Sodium (Na)<br>- Potassium (K)<br>- Chloride (Cl)<br>- Magnesium (Mg)	X[1]<br>X[1]		As per local practice					
**Pregnancy Test**		X[+]						
**QoL:**<br>• QLQ-30, QLQ-CX24	X		3m	Every 6m	Every 6m			
**Resource Utilization:**<br>• EQ-5D	X		3m	Every 6m	Every 6m			
**AE/SAE, if any**		X						

[*] Within 6 weeks prior to randomization
[1] Within 28 days prior to randomization
[#] Optional, as per local practice
[+] Prior to study intervention

Appendix IV: Quality Of Life Instruments

EORTC QLQ-C30 and QLQ-CX24

For the assessment of general quality of life, the EORTC Core questionnaire (QLQ-30 version 3) will be used. This multidimensional cancer specific QoL questionnaire has been developed and tested in many cancer contexts. It has been found to be valid and reliable. It contains five scales: physical, cognitive, emotional, social and role functioning. It has a global health status scale, three symptom scales (pain, fatigue and nausea/vomiting), and six single items (assessing dyspnea, insomnia, loss of appetite, constipation, diarrhea) and perceived financial impact. Most of the items are rated on a 4-point Likert scale, with the exception of the global quality of life scale which is a 7-point scale. For functional and global quality of life, a higher score represents a better level of functioning. For symptom scales and items, a higher score represents a higher level of symptoms and poorer QoL.

The EORTC QLQ-CX24 module will be used to assess the diagnosis-specific QoL issues related to cervix cancer.

Completion of Quality of Life Instruments

Upon confirmation of eligibility and subsequent to inform consent, but preferably prior to randomization, the patient will be asked to complete the QoL questionnaires. It is preferable for the patient to complete the QoL questionnaires prior to randomization, though this is not mandatory. During the first two years of follow-up, the patient will be asked to complete the QoL questionnaires at 3, 6, 12, 18, and 24 months after randomization.

Quality of Life questionnaires are to be completed by the patient. They can be either mailed to the patient or completed by the patient at a clinic visit. The research nurse or delegated personnel at the clinical centre should indicate to the patient that the information the patient is providing is completely confidential. Instructions for the completion of questionnaires:

1. It is completed totally by the patient. Assistance should not be provided from the clinic staff or the patient's relatives unless the patient is blind or illiterate.
2. Only one answer to every question should be checked.
3. Clinical centre personnel should not review the questionnaire with the patient prior to completion.
4. Following completion, the research nurse or delegate must confirm verbally with the patient that the questionnaire has been fully completed. The patient should not change any responses after questionnaire completion.

EORTC QLQ-C30 (version 3)

We are interested in some things about you and your health. Please answer all of the questions yourself by circling the number that best applies to you. There are no "right" or "wrong" answers. The information that you provide will remain strictly confidential.

Please fill in your initials:

Your birthdate (Day, Month, Year):

Today's date (Day, Month, Year): 31

		Not at All	A Little	Quite a Bit	Very Much
1.	Do you have any trouble doing strenuous activities, like carrying a heavy shopping bag or a suitcase?	1	2	3	4
2.	Do you have any trouble taking a long walk?	1	2	3	4
3.	Do you have any trouble taking a short walk outside of the house?	1	2	3	4
4.	Do you need to stay in bed or a chair during the day?	1	2	3	4
5.	Do you need help with eating, dressing, washing yourself or using the toilet?	1	2	3	4

During the past week:

		Not at All	A Little	Quite a Bit	Very Much
6.	Were you limited in doing either your work or other daily activities?	1	2	3	4
7.	Were you limited in pursuing your hobbies or other leisure time activities?	1	2	3	4
8.	Were you short of breath?	1	2	3	4
9.	Have you had pain?	1	2	3	4
10.	Did you need to rest?	1	2	3	4
11.	Have you had trouble sleeping?	1	2	3	4
12.	Have you felt weak?	1	2	3	4
13.	Have you lacked appetite?	1	2	3	4
14.	Have you felt nauseated?	1	2	3	4
15.	Have you vomited?	1	2	3	4
16.	Have you been constipated?	1	2	3	4

Please go on to the next page

**During the past week:**	**Not at All**	**A Little**	**Quite a Bit**	**Very Much**
17. Have you had diarrhea?	1	2	3	4
18. Were you tired?	1	2	3	4
19. Did pain interfere with your daily activities?	1	2	3	4
20. Have you had difficulty in concentrating on things, like reading a newspaper or watching television?	1	2	3	4
21. Did you feel tense?	1	2	3	4
22. Did you worry?	1	2	3	4
23. Did you feel irritable?	1	2	3	4
24. Did you feel depressed?	1	2	3	4
25. Have you had difficulty remembering things?	1	2	3	4
26. Has your physical condition or medical treatment interfered with your family life?	1	2	3	4
27. Has your physical condition or medical treatment interfered with your social activities?	1	2	3	4
28. Has your physical condition or medical treatment caused you financial difficulties?	1	2	3	4

For the following questions please circle the number between 1 and 7 that best applies to you

29. How would you rate your overall health during the past week?

1	2	3	4	5	6	7
Very poor						Excellent

30. How would you rate your overall quality of life during the past week?

1	2	3	4	5	6	7
Very poor						Excellent

© Copyright 1995 EORTC Quality of Life Group. All rights reserved. Version 3.0

EORTC QLQ – CX24

Patients sometimes report that they have the following symptoms or problems. Please indicate the extent to which you have experienced these symptoms or problems, please answer by circling the number that best applies to you.

During the past week:	Not at all	A little	Quite a bit	Very much
31. Have you had cramps in your abdomen?	1	2	3	4
32. Have you had difficulty in controlling your bowels?	1	2	3	4
33. Have you had blood in your stools (motions)?	1	2	3	4
34. Did you pass water/urine frequently?	1	2	3	4
35. Have you had pain or a burning feeling when passing water/urinating?	1	2	3	4
36. Have you had leaking of urine?	1	2	3	4
37. Have you had difficulty emptying your bladder?	1	2	3	4
38. Have you had swelling in one or both legs?	1	2	3	4
39. Have you had pain in your lower back?	1	2	3	4
40. Have you had tingling or numbness in your hands or feet?	1	2	3	4
41. Have you had irritation or soreness in your vagina or vulva?	1	2	3	4
42. Have you had discharge from your vagina?	1	2	3	4
43. Have you had abnormal bleeding from your vagina?	1	2	3	4
44. Have you had hot flushes and/or sweats?	1	2	3	4
45. Have you felt physically less attractive as a result of your disease or treatment?	1	2	3	4
46. Have you felt less feminine as a result of your disease or treatment?	1	2	3	4
47. Have you felt dissatisfied with your body?	1	2	3	4

Please go on to the next page

**During the past 4 weeks:**	**Not at all**	**A little**	**Quite a bit**	**Very much**
48. Have you worried that sex would be painful?	1	2	3	4
49. Have you been sexually active?	1	2	3	4

**Answer these questions only if you have been sexually active during the past 4 weeks:**	**Not at all**	**A little**	**Quite a bit**	**Very much**
50. Has your vagina felt dry during sexual activity?	1	2	3	4
51. Has your vagina felt short?	1	2	3	4
52. Has your vagina felt tight?	1	2	3	4
53. Have you had pain during sexual intercourse or other sexual activity?	1	2	3	4
54. Was sexual activity enjoyable for you?	1	2	3	4

© QLQ-CX24 Copyright 2003 EORTC Quality of life Group. All rights reserved. (phase III module)

Appendix V: Health Utility Instrument

Completion of Health Utility Instrument

Upon confirmation of eligibility and subsequent to inform consent, but preferably prior to randomization, the patient will be asked to complete the EQ-5D health utility questionnaire. It is preferable for the patient to complete the EQ-5D questionnaire prior to randomization, though this is not mandatory. During the first two years of follow-up, the patient will be asked to complete the EQ-5D questionnaire at 3, 6, 12, 18, and 24 months after randomization.

EQ-5D Health Questionnaire

(Canadian English Version)

By placing a check-mark in one box in each group below, please indicate which statements best describe your own state of health today.

Mobility

I have no problems in walking about ❑

I have some problems in walking about ❑

I am confined to bed ❑

Self-Care

I have no problems with self-care ❑

I have some problems washing or dressing myself ❑

I am unable to wash or dress myself ❑

Usual Activities (*e.g. work, study, housework, family or leisure activities)*

I have no problems with performing my usual activities ❑

I have some problems with performing my usual activities ❑

I am unable to perform my usual activities ❑

Pain/Discomfort

I have no pain or discomfort ❑

I have moderate pain or discomfort ❑

I have extreme pain or discomfort ❑

Anxiety/Depression

I am not anxious or depressed ❑

I am moderately anxious or depressed ❑

I am extremely anxious or depressed ❑

Best
imaginable
state of health

100

To help people say how good or bad their state of health is, we have drawn a scale (rather like a thermometer) on which the best state you can imagine is marked 100 and the worst state you can imagine is marked 0.

We would like you to indicate on this scale how good or bad your own health is today, in your opinion. Please do this by drawing a line from the box below to whichever point on the scale indicates how good or bad your state of health is today.

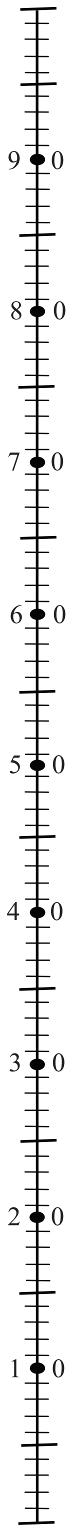

Your own state of health today

0

Worst
imaginable
state of health

©1990 EuroQol Group EQ-5D™

Appendix VI: ^{18}FDG PET-CT + Contrast-enhanced CT Abdomen and Pelvis Scanning Procedure

Figure 1. Timeline of Patient Preparation for PET-CT +CT Contrast-enhanced CT-Integrated to PET/CT Imaging

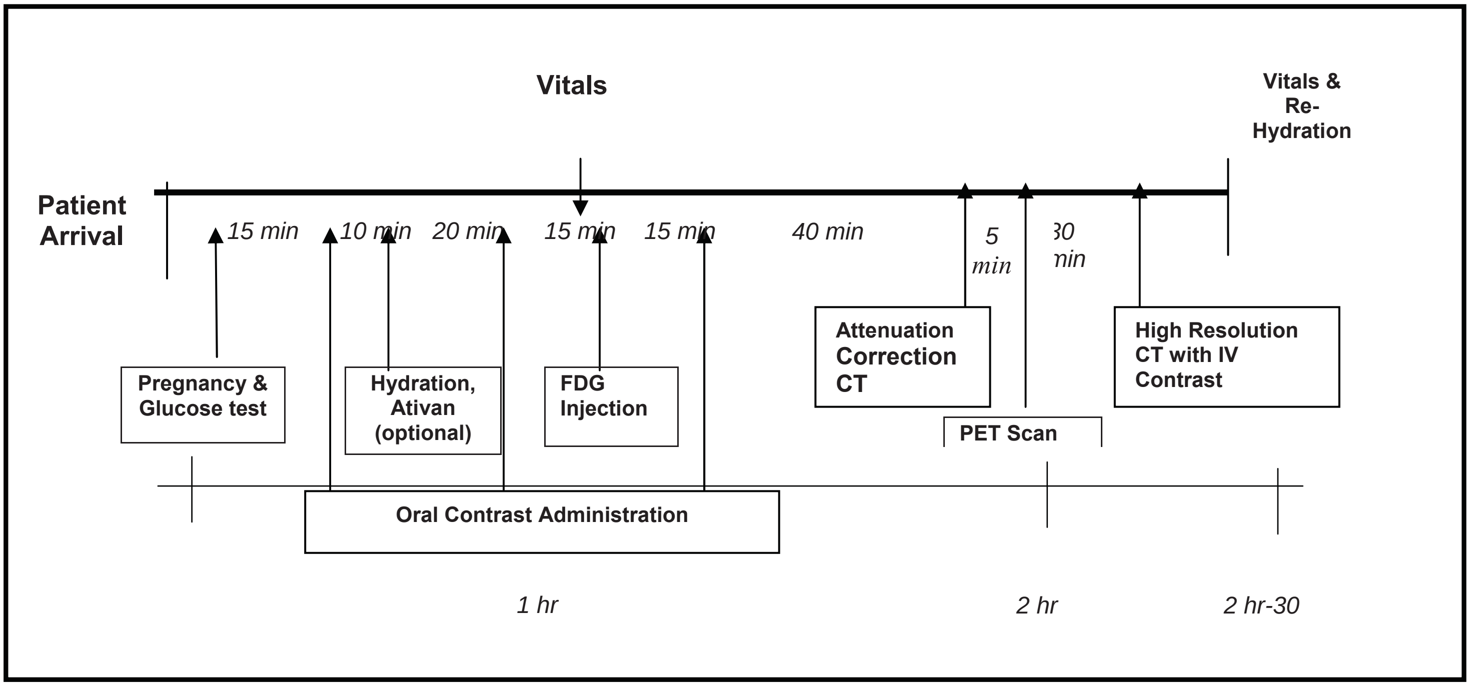

Note: If contrast-enhanced CT of abdomen and pelvis has been done prior to randomization, then a low resolution CT is done with the PET. If a contrast-enhanced CT of abdomen and pelvis was performed within 6 weeks prior to randomization, it is not repeated.

1. *Instructions for Patient when Booking PET Scan*

a) Exercise Instructions
Patients should refrain from vigorous exercise 48 hours prior to the test.

b) Fasting Instructions
Patients must fast for a minimum of 4-6 hours but are able to drink water. Patients must be off dextrose or lactose-containing IV for 4 hours. They should also take their usual medications. For scans performed in the morning, the patient will be NPO from midnight. For studies performed in the afternoon, patients will be allowed a light breakfast followed by a 4 hour fast.

c) Insulin Protocol
All insulin dependent diabetics will be studied in the early afternoon following a light high-protein/low-carbohydrate breakfast and the routine morning administration of insulin. There will be a minimum of a 3-hour wait following the last administration of insulin.

d) **Discussion with Patient**
Answer any questions the patient might have and determine any further issues that may need to be addressed prior to scan date.

2. Patient Preparation on Day of the PET Scan

a) **Patient Arrival**
Patients are to arrive between 45-60 minutes before their planned FDG injection time. Patient should arrange a companion to escort them after the scan.

b) **Patient Hydration**
When patient arrives, provide a bottle of water to drink while waiting.

c) **Blood Glucose Testing**
Blood glucose levels will be measured prior to administration of FDG. If the serum glucose is greater than 9.7 mmol/L (175 mg/dl), the scan will be rebooked. If necessary, the patient will be referred to the physician responsible for his/her diabetic management to adjust medication and diet until the fasting blood glucose has been demonstrated to be less than 9.7 mmol/L on two occasions.

d) **Pregnancy Testing**
Women with childbearing potential must have a blood or urine pregnancy test prior to the PET scan and the results must be known prior to the FDG injection. If the patient is pregnant, they must not undergo PET scanning. Ensure that the patient is not breast-feeding a baby.

e) **Ativan Administration**
Ativan 50 µg/kg, sublingually, to a maximum of 2 mg may be administered at the discretion of the supervising physician, 30 to 60 minutes prior to FDG injection. This will encourage muscular relaxation and reduce muscle uptake.
Patient should be kept warm and rest quietly while waiting for their scan.

f) **Reporting of Concomitant Medication**
All medications being taken by the patient at the time of the PET scanning will be recorded on the *PET Scan Module* Case Report Form.

3. Administration of FDG

a) **Patient Measurement**
Have patient change into a gown, removing all metal objects including bra. Measure the patient’s height and weight.

b) **Vitals**
Heart rate, blood pressure and temperature will be taken prior to FDG injection.

c) **FDG Calibration**
The injection syringe will be calibrated pre- and post-injection to enable calculation of the injected dose.

d) FDG Injection
The patient will be injected with 5MBq/kg of ^{18}FDG to a maximum of 500 MBq. The site of the injection will be recorded. If the FDG injection site is not included in the field of view, it is recommended that a radiation monitor be used to detect if any extravasation occurred. Report any problems with injection on the *PET Scan Module* Case Report Form.

e) Patient Hydration
Patients will be well hydrated and, where feasible, will drink 500 ml of water after injection.

f) Furosemide Administration
At the discretion of the supervising physician, furosemide (20 mg) may be injected within 10 minutes following ^{18}FDG injection to promote diuresis.

g) Patient Resting Prior to Scan
The patient will then rest in a quiet room in order to decrease any physiological muscular uptake of FDG prior to PET imaging.

4. *Patient Positioning*

Patients do not have to be imaged with their arms up, although this is preferred, and do not have to be imaged on a flat PET/CT couch insert.

5. *Field of View*

The same field of view should be used for both CT and PET imaging. Image from the base of the skull down to the mid thigh.

6. *Contrast-enhanced CT Scanning Procedures*

a) CT performed on separate instrumentation
Since PET scanning units and procedures vary at study centres, contrast-enhanced CTs can be performed on either PET scanning units or CT units. In centres with a 16 or higher slice PET scanning unit, the PET-CT and CT of the abdomen and pelvis can be performed as a one step procedure as outlined in Figure 1. In those centres with lower (i.e. 2 slice PET scanning units), the PET-CT is generally performed first on a PET scanning unit followed by a high resolution CT performed the same day on a CT unit.

b) Oral Contrast
Diluted oral contrast will be administered. The recommended administration is 20cc of Telebrix or Gastrograffin diluted in water to a total volume of 1L (1000 cc), to be given to patients over one hour prior to the CT or PET/CT scan. 750 cc should be administered an hour before the scan and the remaining 250 cc just before the CT or PET/CT scan.

c) CT Timing
Refer to Figure 1 for timing of examinations.

d) Scan Parameters
As per the clinical centre's standard protocol.

e) **Angulation**
No gantry angulation is to be utilized.

f) **Slices**
As per the clinical centre's standard protocol.

7. PET Scanning Procedures

Scanning will be initiated one hour post FDG injection with collection of an emission image of the body from the base of the skull through to the mid thigh. Images will be reconstructed for review both with and without attenuation correction. Patient positioning and field of view should be identical to CT imaging.

8. PET Data Acquisition

Acquire clinically acceptable scans in 2D or 3D mode.

9. Post-Scan Procedure

a) **Vitals**
Heart rate, blood pressure and temperature will be taken post-scan.

b) **Adverse Event Reporting**
If the patient experiences any adverse events related to the scanning procedure, record the events on the *Adverse Event* Case Report Form.

c) **Rehydration**
Patients should be offered juice or water if necessary.

APPENDIX VII: ^{18}FDG PET IMAGE INTERPRETATION

To ensure quality control of PET interpretation, a sample of PET scans will be independently interpreted by a primary nuclear medicine physician at the enrolling Clinical Centre and a secondary nuclear medicine physician from an external centre with compatible PET equipment.

Only the primary reading will affect patient management. The secondary reading will be performed on digital images transferred by compact disc. The secondary readers will be provided with the data required for scanning (e.g. height and weight) and scanning details (e.g. injected FDG dose), as well as a short description of relevant clinical findings important for interpretation of the PET results, prepared by the local PET Reader (e.g., information about recent surgery that may be similar in appearance to a tumour on the PET image).

Whole body PET-CT scans will be interpreted based on attenuation-corrected and non-attenuation-corrected results. The reader's degree of suspicion for an abnormality will be recorded as per image interpretation guidelines specified in section 7.3 with use of a **5-point ordinal categoric scale**, with the following categories: 0-normal, 1-probably normal, 2-equivocal, 3-probably abnormal and 4-definitely abnormal.

For all lesions, semi-quantitative measurement of the **single-pixel maximal standard uptake value normalized to body mass** will be performed on the primary tumour. The SUV_{BM} is calculated using the tumour radioconcentration Q (Bq/cc), body mass m (kg) and injected activity Q_{inj} (Bq):

$$SUV_{BM} = (Q / Q_{inj}) \times m \times 1000$$

The transaxial slice number that contains the single pixel maximal SUV will also be identified. **SUV should be measured with an ROI to encompass at most 2/3 of the diameter of the node to minimize partial volume effects.**

As there may be variability in SUV measurements between different PET centres and between different studies, 2 measurements of SUV of the liver should also be recorded as normal reference. A 3 cm diameter region of interest in the right lobe of the liver should be used for these 2 measurements.

Recorded data will be transmitted to OCOG either on paper case report forms or through EDC. OCOG will evaluate both interpretations and determine if there are any major discrepancies. Examples of major discrepancies are (a) a difference in the location of abnormality and (b) a difference of more than 1 point in the 5-point ordinal categorical scale (e.g. 2-equivocal vs. 4-definitely abnormal).

Major discrepancies between the primary and secondary readers will be resolved by consensus between the two readers. Meetings to discuss scans will be coordinated by OCOG

APPENDIX VIII: AMINOGLYCOSIDE ANTIBIOTICS

Generic	***Brand Name***
Amikacin	*Amikin*
Gentamicin	*Garamycin, G-Mycin, Jenamicin*
Kanamycin	*Kantrex*
Neomycin	*Mycifradin, Myciguent*
Netilmicin	*Netromycin*
Paramomycin	
Streptomycin	
Tobramycin	*Nebcin*

APPENDIX IX: ECONOMIC ANALYSIS

Economic Evaluation

Costs

We have recently worked with Decision Support Services at Hamilton Health Sciences to successfully obtain costs for treatments, tests, medications, hospitalizations from the Ontario Case Costing Data Base for two of OCOG's other PET trials in lung cancer and breast cancer.[45] We will use the same approach for this trial for costs related to hospitalizations, tests, procedures, medications and the use of other healthcare services. Costs related to physician services will be taken from the recent OHIP payment schedule.[46] For chemotherapy costs we will employ a bottom up costing approach.[47] For radiotherapy costs, we will review funding relationships between individual cancer centres, CCO and the MOHLTC to determine appropriate costs. For example, currently the MOHLTC reimburses cancer centres $3500 per new patient radiated.

Total healthcare cost including all imaging and treatment will be calculated for each patient and averaged across patients in each study arm. Once patients develop recurrence of their cancer, treatments will vary. We will capture what treatments were received and obtain costs estimates for these but further data collection will not take place.

In addition to the two cancer specific QoL questionnaires, utilities will be estimated using the EQ-5D measurement system. The EQ-5D is a reliable and valid questionnaire designed to estimate societal valuations of the quality of life of patients (utilities).[48,49] For this analysis, the UK tariff for estimation of utilities will be adopted.[50] The EQ5D will be used to estimate utility values at each assessment point. These will then be aggregated using area under the curve methodology controlling for baseline utility value to allow estimation of quality adjusted life years (QALYs).

Analysis

Analysis will take the form of a traditional concurrent clinical and economic evaluation with prospective follow-up of patients. The economic evaluation will follow Canadian guidance on the conduct of such studies.[51] Analysis will take the form of both a cost effectiveness and a cost utility analysis.

For the cost effectiveness analysis, effectiveness will be assessed by the primary outcome of the clinical trial; the treatment delivered based on an imaging strategy. The analysis will assess the incremental costs associated in delivering either chemo-radiotherapy with extended field radiation or avoiding radical chemo-radiotherapy of curative intent and proceeding to palliation or non-curative chemotherapy or radiation versus standard curative chemo-radiotherapy. This will be estimated by:

$$\frac{C_{PET\text{-}CT} - C_{CT}}{E_{PET\text{-}CT} - E_{CT}}$$

Where:

$\underline{C_{PET\text{-}CT}}$ = average total costs for patients randomized to the CT abdomen and pelvis plus PET-CT arm – including all imaging and subsequent treatment

$\underline{C_{CT}}$ = average total costs for patients randomized to the CT abdomen and pelvis arm

$\underline{E_{PET\text{-}CT}}$ = Probability of change in management based on an imaging strategy for patients randomized to the CT abdomen and pelvis plus PET-CT arm

$\underline{E_{CT}}$ = Probability of change in management based on an imaging strategy for patients randomized to the CT abdomen and pelvis arm

For the cost utility analysis effectiveness will be assessed by QALYs as described in the Quality of Life analysis section. This will be estimated by:

$$\frac{C_{PET\text{-}CT}-C_{CT}}{QALY_{PET\text{-}CT}-QALY_{CT}}$$

Where:

$QALY_{PET\text{-}CT}$ = Average total QALYs for patients randomized to the CT abdomen and pelvis plus PET-CT arm

$QALY_{CT}$ = Average total QALYs for patients randomized to the CT abdomen and pelvis arm

Time Horizon

Primary analysis will have a time horizon of two years reflective of the period of data capture within the clinical trial. Secondary analysis will involve forecasting costs and utility values up to a time horizon of ten years. Costs and benefits will be discounted at 5%.

Perspective

Analysis will be conducted from the healthcare system perspective.

Analysis of Uncertainty

The uncertainty concerning the incremental cost, the incremental effectiveness and the incremental cost-effectiveness ratio will be estimated by conducting probabilistic analysis through non-parametric bootstrapping.[52] Bootstrapping allows estimation of the dispersion around an outcome of interest. With bootstrapping, the study sample is treated as the patient population. We then re-estimate the study sample through drawing repeated random samples of the same size as the original sample. This is done by drawing individual patient samples with replacement from the original data.

For this study, we will obtain 5,000 estimates of costs and effectiveness (each of the primary and secondary outcomes) for each strategy. This approach will be used to derive 95% certainty intervals around the difference in costs, outcomes and, where pertinent, the incremental cost per outcome. These certainty intervals will be estimated using the bias corrected percentile method.[53]

Results from the bootstrapping exercise will also be used to depict cost effectiveness acceptability curves (CEACs). CEACs are a graphical representation of the probability that a treatment may be cost effective given alternate dollar values placed on an outcome.[54] This will allow estimation of the probability that the experimental treatment can be considered cost effective given the available data.
