# Supplementary material for: Effect of Positron Emission Tomography Imaging in Women With Locally Advanced Cervical Cancer: A Randomized Clinical Trial
Source: JAMA Netw Open. 2018 Sep 14;1(5):e182081. doi: 10.1001/jamanetworkopen.2018.2081 (PMC6324512; doi:10.1001/jamanetworkopen.2018.2081)
Supplement: Supplement 2. — eTable 1. Radiotherapy Guidelines eAppendix. Sample Size Calculation eTable 2. Lymph Nodes, PET-CT, and Treatment Delivered eTable 3. Lymph Nodes, CT, and Treatment Delivered [file jamanetwopen-1-e182081-s002.pdf]

Supplementary Online Content

Elit LM, Fyles AW, Gu C-S, et al. Effect of Positron Emission Tomography Imaging in Women With Locally Advanced Cervical Cancer: A Randomized Clinical Trial. *JAMA Netw Open.* 2018;1(5):e182081. doi:10.1001/jamanetworkopen.2018.2081

eTable 1. Radiotherapy Guidelines

eAppendix. Sample Size Calculation

eTable 2. Lymph Nodes, PET-CT, and Treatment Delivered

eTable 3. Lymph Nodes, CT, and Treatment Delivered

This supplementary material has been provided by the authors to give readers additional information about their work.

eTable 1: Radiotherapy Guidelines

Imaging	Recommended treatment	
Disease in Para-aortic nodes	EFRT	Extend radiation field to include Para-aortic nodes to T12/L1
Disease identified in pelvic nodes or presacral nodes		Extend superior pelvic margin to include common iliac nodes to L4 and/or include whole sacrum
Disease identified in common iliac nodes		Extend superior pelvic field margin to include low Para-aortic nodes i.e., L1/2
Extensive disease identified in primary cervix tumor or pelvic nodes	Modify pelvic radiation fields without EFRT	
More extensive cervical disease e.g. uterine extension	Change brachytherapy volume or dose	
Supraclavicular nodes, mediastinal nodes, bony mets, other solid organ disease	Palliative care only i.e., chemo alone or palliative radiation therapy, clinical trials or supportive care	

© 2018 Elit LM et al. *JAMA Network Open.*

eAppendix: Sample Size Calculation

It was hypothesized that 25% of patients having a CT of the abdomen and pelvis alone would receive either palliative treatment (10%) or EFRT (15%). The use of PET CT in addition to CT abdomen and pelvis was to be considered of clinical interest if the rate of treatment with either palliation or EFRT increased to 45% (20% and 25%, respectively). In an attempt to improve accrual, a 2:1 allocation ratio favoring PET-CT was implemented. Given the complexities involved in valid assumptions for calculating power using a logistic regression model accounting for stratum, sample size calculations were based on an $\alpha=0.05$, two-sided, Fisher's exact test.

© 2018 Elit LM et al. *JAMA Network Open.*

eTable 2: Lymph Nodes, PET-CT, and Treatment Delivered

Lymph Nodes Abnormal			No. of Patients	Radiation Delivered		
Pelvic	Common Iliac	Para-aortic		Standard	More Extensive	Palliative
**+**	**+**	**+**	3	0	2	1
**+**	**+**	**-**	8	2	5	1
**+**	**-**	**+**	8	2	5	1
**+**	**-**	**-**	17	11	5	2
**-**	**+**	**-**	2	1	1	0
**-**	**-**	**+**	6	2	4	0
**-**	**-**	**-**	58	45	12	1
**-**	**+**	**+**	1	0	1	0

+ reflects abnormal lymph nodes

© 2018 Elit LM et al. *JAMA Network Open.*

eTable 3: Lymph Nodes, CT, and Treatment Delivered

Lymph Nodes Abnormal			Patient No.	Radiation Delivered			
Pelvic	Common Iliac	Para-aortic		Standard	More Extensive	Palliative	Other
**+**	**+**	**+**	2	0	0	2	0
**+**	**+**	**-**	1	1	0	0	0
**+**	**-**	**-**	`13	8	5	0	0
**-**	**+**	**-**	2	0	2	0	0
**-**	**-**	**+**	3	1	2	0	0
**-**	**-**	**-**	34	30	2	1	1

+ reflects abnormal lymph nodes

© 2018 Elit LM et al. *JAMA Network Open.*
